# Expression of Toll-like Receptors on Lymphocyte Subpopulations and Their Soluble Forms in Serum and Urine of Women with Endometriosis

**DOI:** 10.3390/cells14161273

**Published:** 2025-08-18

**Authors:** Anna Sobstyl, Paulina Mertowska, Sebastian Mertowski, Rafał Tarkowski, Dominik Dudziński, Michał Kotowski, Krzysztof Bojarski, Bogusława Stelmach, Błażej Chermuła, Maciej Brązert, Ewelina Grywalska

**Affiliations:** 1 Department of Experimental Immunology, Medical University of Lublin, Chodźki 4a St., 20-093 Lublin, Poland; sobstylanna.1@gmail.com (A.S.); d.dudzinski1996@gmail.com (D.D.); ewelina.grywalska@umlub.pl (E.G.); 2 I Chair and Department of Oncological Gynaecology and Gynaecology, Medical University of Lublin, 20-059 Lublin, Poland; rafal.tarkowski@umlub.pl; 3 Chair and Department of Paediatric Otolaryngology, Phoniatry and Audiology, Medical University of Lublin, Antoniego Gębali 6 Street, 20-093 Lublin, Poland; michal.kotowski@umlub.pl; 4 General Surgery Department, SP ZOZ in Leczna, 52 Krasnystawska Street, 21-010 Leczna, Poland; k.bojarski@szpital.leczna.pl; 5 Department of Diagnostics and Treatment of Infertility, Poznan University of Medical Sciences, 61-701 Poznan, Poland; b_stelmach@wp.pl (B.S.); blazej.chermula@wp.pl (B.C.); maciejbrazert@ump.edu.pl (M.B.)

**Keywords:** endometriosis, Toll-like receptors (TLRs), innate immunity, immune dysregulation, lymphocytes, TLR expression, biomarkers, chronic inflammation, immunopathogenesis

## Abstract

Introduction: Endometriosis is a chronic inflammatory disease affecting women of reproductive age, often accompanied by chronic pelvic pain and infertility. Despite numerous studies, its pathogenesis remains incompletely understood. Increasing evidence indicates the important role of immunological disorders, especially in the mechanisms of innate immunity and Toll-like receptors (TLRs). Study objective: This study aimed to assess the expression of selected TLRs (TLR2, TLR3, TLR4, TLR7, TLR8, and TLR9) on peripheral blood lymphocyte subpopulations (CD4+, CD8+, and CD19+ cells) in patients diagnosed with endometriosis and to quantify the levels of their soluble forms in serum and urine. This study was conducted on patients who were not undergoing hormonal bridging therapy and were not using any form of hormonal contraception to eliminate potential confounding effects on immune parameters. Methods: Flow cytometric analysis of TLR expression on peripheral blood lymphocytes was performed, and the levels of their soluble forms in serum and urine samples were determined. Additionally, ROC curve analysis was used to evaluate the diagnostic potential of the studied parameters. Results: We found increased expression of TLRs in lymphocyte populations in patients with endometriosis compared to the control group, suggesting their involvement in both local and systemic immune responses. In addition, ROC analysis showed the diagnostic potential of TLR expression in differentiating patients with endometriosis from healthy women, and it may also identify disease subtypes. Conclusions: The findings support the role of TLRs in the immunopathogenesis of endometriosis and highlight their promise as diagnostic biomarkers and therapeutic targets. Further studies on larger patient cohorts and functional signaling analyses are warranted.

## 1. Introduction

Endometriosis is a chronic, estrogen-dependent gynecological disease with complex etiopathogenesis, affecting approximately 10% of women of reproductive age, of whom up to 70% experience chronic pelvic pain and fertility disorders [1,2,3,4]. This disease is characterized by the presence of active endometrial foci outside the uterine cavity, which leads to a persistent inflammatory reaction, angiogenesis, fibrosis, and damage to the surrounding tissues [5,6]. Despite numerous studies, the molecular mechanisms underlying the development and progression of endometriosis remain incompletely understood. Current data indicate that dysregulation of the immune system plays a key role in the pathogenesis of the disease, both in the innate and acquired responses [7,8,9,10,11,12]. In particular, abnormalities in the functioning of T lymphocytes (especially the Th17 and Treg subpopulations), B lymphocytes, impaired phagocytosis by macrophages, and reduced cytotoxic activity of NK cells have been described, which result in the ineffective elimination of endometrial cells from ectopic locations.

Among the molecular mediators of the nonspecific response, an important role is attributed to Toll-like receptors (TLRs), which serve as key sensors of pathogens and signals of endogenous threats. The expression of TLR2, TLR3, TLR4, and TLR9 has been demonstrated in ectopic endometrium foci, in stromal and epithelial cells, and in infiltrating macrophages [13,14,15,16,17]. Their activation initiates signaling cascades dependent on the MyD88 and TRIF adaptors, leading to the activation of the transcription factor NF-κB and increased production of pro-inflammatory cytokines (IL-6, IL-8, TNF-α). Chronic, sterile TLR stimulation—induced, for example, by endogenous DAMPs or microbiome components—can sustain inflammation, promote angiogenesis, and support the survival of endometrial cells [14,15,18,19]. Additionally, disturbances in the negative regulation of these pathways have been observed, contributing to persistent low-intensity inflammation. Increasing importance is also attributed to interactions between TLRs and the intestinal and endometrial microbiota, which can modulate the local immune response and affect immunotolerance [20,21,22,23,24,25,26,27,28].

Immune regulation in women is closely related to the action of sex hormones, mainly estrogens and progesterone. Estrogens enhance the activation of immune cells, increasing the production of pro-inflammatory cytokines and the expression of TLRs, especially TLR2 and TLR4. At the same time, progesterone has an inhibitory effect, promoting tolerance and limiting the activation of the NF-κB pathway [29,30,31,32,33,34]. In patients with endometriosis, a disturbance of this balance is observed—estrogen dominance and progesterone resistance, which promote the perpetuation of inflammation and the pathological stimulation of the immune system, including excessive activation of TLRs. These complex hormonal–immunological relationships, further reinforced by oxidative and microbiological factors, promote the survival, migration, and invasiveness of endometrial cells, thereby facilitating the clinical progression of the disease.

A review of the scientific literature available in the PubMed database indicates that out of 37,427 publications containing the term “endometriosis” (1927–2025), only about 4% (approx. 1482 works) directly concern the involvement of the immune system, and the issue related to TLRs is addressed by only 0.12% of available publications. This state of affairs highlights a significant research gap in the immunobiology of endometriosis, with particular emphasis on the innate response and the role of TLRs in the molecular mechanisms of this disease.

In light of the above, this study aimed to assess the expression of selected TLRs (TLR2, TLR3, TLR4, TLR7, TLR8, and TLR9) on subpopulations of T lymphocytes (CD4+ and CD8+) and B lymphocytes (CD19+) in the peripheral blood of patients with newly diagnosed, untreated endometriosis. Additionally, an analysis of their soluble form concentrations in serum and urine was performed and compared to the control group. The key methodological assumption of this study was to exclude patients using any hormonal therapy, including hormonal contraception or bridging treatment. Thanks to this, the influence of exogenous hormones on immunological parameters was eliminated, allowing for a more objective picture of the natural immune mechanisms associated with endometriosis and a more accurate assessment of the potential role of TLRs in the disease’s pathogenesis.

## 2. Materials and Methods

### 2.1. Patient Characteristics

This study included a total of 74 women, comprising 50 patients diagnosed with endometriosis and 24 individuals without endometriosis, who served as the control group. The diagnosis of endometriosis was established based on the criteria of the American Society for Reproductive Medicine (ASRM) [35] and the clinical guidelines of the Polish Society of Gynecologists and Obstetricians [36] for the management of women with endometriosis. The control group included women undergoing surgery for benign ovarian cysts, diagnostic laparoscopy, or tubal ligation, as well as healthy volunteers (HV).

Inclusion criteria for the study group were as follows:

- Confirmed diagnosis of endometriosis by histopathological and clinical evaluation;
- Age ≥ 18 years;
- No immunosuppressive treatment within the past 12 months;
- No use of hormonal contraception (including intrauterine systems);
- Provision of written informed consent to participate in the study.

Exclusion criteria were as follows:

- Presence of uterine fibroids or adenomyosis;
- Active bacterial, viral, or fungal infections;
- Diabetes mellitus or other metabolic/endocrine disorders;
- Chronic wounds, severe allergic diseases, or significant systemic illnesses affecting the kidneys, gastrointestinal tract, heart, or lungs, as well as adherence to strict elimination diets;
- History of hematopoietic stem cell or organ transplantation;
- Active malignancy or autoimmune disorders;
- Pregnancy or breastfeeding;
- Use of immunosuppressive medications or participation in other clinical trials;
- Presence of CNS metastatic disease or diagnosed psychiatric disorders.

An experienced gynecologist specializing in the diagnosis and treatment of endometriosis established the diagnosis. Initial diagnostic steps included transvaginal and transabdominal ultrasound examinations of the pelvis and other anatomical sites suspected of endometrial lesions. In more complex cases, additional imaging with computed tomography (CT) or magnetic resonance imaging (MRI) was performed to enhance diagnostic accuracy. The final diagnosis was confirmed through histopathological evaluation of tissue samples obtained during laparoscopy.

In the control group, no evidence of ectopic endometrial tissue was identified in pelvic or abdominal ultrasound imaging. From each participant, peripheral blood (10 mL) was collected via the basilic vein into EDTA-coated tubes (Sarstedt, Nümbrecht, Germany) for immunophenotyping. An additional 5 mL of blood was obtained using tubes (Sarstedt, Nümbrecht, Germany) with a clot activator for serum isolation and analysis of soluble TLRs. Furthermore, 10 mL of midstream urine was collected for analysis of urinary biomarkers. All biological samples were obtained during the follicular phase of the menstrual cycle (days 5–11) to minimize hormonal variability. Based on the location and type of endometrial lesions—composed of ectopic endometrial glands and stroma—patients diagnosed with endometriosis were classified into the following subgroups:

- Peritoneal endometriosis (PE);
- Ovarian endometriosis (OE);
- Deeply infiltrating endometriosis (DIE);
- Cesarean section scar endometriosis (CC).

This stratification was used in subsequent analyses to evaluate potential immunological and molecular differences across disease subtypes.

Basic blood parameters were determined from fasting venous blood collected into two types of tubes: one with EDTA (for complete blood count) (Sarstedt, Nümbrecht, Germany) and one with a clot activator (Sarstedt, Nümbrecht, Germany) (for CRP and immunoglobulins). Complete blood counts, including RBC [M/μL], WBC [K/μL], LYM [K/μL], MON [K/μL], NEU [K/μL], EOS [K/μL], BAS [K/μL], HGB [g/dL], and PLT [K/μL], were determined by the optical impedance method using an automated hematology analyzer (Sysmex XN-1000, Sysmex Corporation, Kobe, Japan) employing standard laboratory procedures. CRP concentration [mg/L] was determined in serum by the immunoturbidimetric method on a biochemistry analyzer (Cobas, Abbott, Chicago, IL, USA). IgG, IgA, and IgM immunoglobulin levels [g/L] were determined by the nephelometric method using an immunochemical analyzer (BN ProSpec System, Siemens Healthineers, Marburg, Germany). All tests were performed at the time of patient recruitment in the hospital laboratory.

The study was conducted in accordance with the Declaration of Helsinki and approved by the Bioethics Committee of the Medical University of Lublin (KE-0254/10/01/2023). All participants signed informed consent and were informed of the nature and purpose of the study.

### 2.2. Immunophenotyping

Phenotypic characterization of peripheral blood lymphocytes was conducted using multiparameter flow cytometry. For surface and intracellular staining, a panel of monoclonal antibodies specific to selected lineage markers and TLRs was employed. Detailed information regarding the antibodies used is provided in Table S1 of the Supplementary Materials.

To minimize spectral overlap when using fluorochromes excited by violet lasers, BD Horizon™ Brilliant Stain Buffer Plus (BD Biosciences Franklin Lakes, NJ, USA, Cat. No. 566385) was incorporated into the antibody cocktail, as recommended by the manufacturer. After adding the antibodies, samples were incubated for 20 min at room temperature in the dark. Subsequently, cells were washed using BD Pharmingen™ Stain Buffer (FBS) (BD Biosciences, Franklin Lakes, NJ, USA Cat. No. 554656) to remove unbound antibodies.

For the intracellular staining of TLRs, the BD Cytofix/Cytoperm™ Fixation/Permeabilization Kit (BD Biosciences, Franklin Lakes, NJ, USA Cat. No. 554714) was employed according to the manufacturer’s protocol, ensuring adequate cell fixation and membrane permeabilization.

Flow cytometric acquisition was performed using the CytoFLEX LX platform (Beckman Coulter, Brea, CA, USA), and data were analyzed with Kaluza software v 2.1 (Beckman Coulter, Brea, CA, USA). Daily instrument performance was validated with CytoFLEX Daily QC Fluorospheres (Beckman Coulter, Brea, CA, USA, Cat. No. C65719). Compensation of spectral overlap across fluorochrome channels was conducted using the VersaComp Antibody Capture Kit (Beckman Coulter, Brea, CA, USA Cat. No. B22804).

To ensure accurate gating and minimize false-positive signals, fluorescence-minus-one (FMO) controls were prepared individually for each anti-TLR antibody. Each FMO sample was derived from the same biological specimen as the fully stained sample and underwent identical staining, fixation, and analysis procedures. Gates for marker positivity were set based on these FMO controls, accounting for background fluorescence and autofluorescence specific to each detector channel.

A detailed gating strategy and representative plots are provided in the Supplementary Materials (Figures S1–S10).

### 2.3. Determination of the Concentration of Soluble Forms of TLRs in the Tested Material

The concentration of soluble forms of Toll-like receptors (sTLRs) was analyzed in serum and urine samples collected from patients with endometriosis and the control group. Serum was obtained by centrifugation of whole blood collected in a clot tube (10 min, 2000× *g*, 4 °C) and then stored at −80 °C until analysis. Urine samples were centrifuged (10 min, 3000× *g*, 4 °C) to remove cell sediment, and the supernatant was frozen at −80 °C. Commercially available immunoenzymatic ELISA kits (Thermo Fisher Scientific, Waltham, MA, USA and Wuhan Fine Biotech Co., Ltd., Wuhan, China) were used for the determinations according to the protocols provided by the manufacturers. Soluble TLR2 and TLR4 concentrations were measured using Invitrogen assays (Thermo Fisher Scientific, Waltham, MA, USA), with a sensitivity of 0.32 ng/mL (TLR2, Cat. No. EH459RB) and 0.4 ng/mL (TLR4, Cat. No. EH460RB), respectively. TLR3 (Cat. No. EH0301; with a sensitivity of 0.094 ng/mL), TLR7 (Cat. No. EH2015; with a sensitivity of 0.094 ng/mL), TLR8 (Cat. No. EH4931; with a sensitivity of 0.094 ng/mL), and TLR9 (Cat. No. EH1019; with a sensitivity of 0.094 ng/mL) were measured using FineTest kits (Wuhan Fine Biotech Co., Ltd., Wuhan, China). Absorbance was measured at the appropriate wavelengths using a VICTOR3™ microplate reader (PerkinElmer, Waltham, MA, USA) and dedicated software.

### 2.4. Statistical Analyses

The data obtained in the study were statistically analyzed using GraphPad Prism (GraphPad Software, v. 9.4.1, San Diego, CA, USA) and Statistica (TIBCO Software, v. 13.3 Palo Alto, CA, USA). Before the analysis, the normality of the variable distributions was assessed using the Shapiro–Wilk test. Due to the lack of compliance with the normal distribution for most of the analyzed parameters, nonparametric statistical tests were used. The Mann–Whitney U test compared two groups (patients with endometriosis vs. the control group), whereas the analysis of differences among more than two groups was performed using the Kruskal–Wallis test, followed by a post hoc test.

A receiver operating characteristic (ROC) curve analysis was performed to evaluate the diagnostic potential of the studied parameters. The analysis was performed using GraphPad Prism (GraphPad Software, v. 9.4.1, San Diego, CA, USA). ROC curves were constructed by plotting the relationship between sensitivity and 1-specificity for different threshold values of the biomarkers studied. The results were visualized as ROC plots indicating the AUC (Area Under the Curve) values.

## 3. Results

### 3.1. Comparison of Immunological and Biochemical Parameters Between Patients with Endometriosis and Healthy Women

The state of chronic inflammation associated with endometriosis affects several hematological, biochemical, and immunological parameters, modifying both local and systemic inflammatory responses and lymphocyte activity [9,36]. Therefore, the first stage of this study involved analyzing differences in basic peripheral blood parameters, inflammatory markers, and subpopulations of immune cells between women with endometriosis (EM) and healthy volunteers (HV).

A total of 74 women were included in the study: 50 patients with newly diagnosed, clinically and histopathologically confirmed endometriosis (according to current diagnostic recommendations; details in Section 2), with a median age of 35 years (range, 24–47 years), and 24 healthy women in the control group, with a median age of 33 years (range, 18–57 years). All participants—both patients and the control group—did not use any form of hormonal therapy, including hormonal contraception, which allowed avoiding the influence of exogenous hormonal modulation on the assessed immunological parameters.

Particular attention was paid to the concentrations of C-reactive protein (CRP), selected immunoglobulins (IgG, IgA, and IgM), and the subpopulation composition of leukocytes, with particular emphasis on T lymphocytes (CD3+, CD4+, and CD8+), B lymphocytes (CD19+), and NK cells. These parameters are crucial in assessing the ongoing inflammation, immune system activity, and potential mechanisms of impaired immunoregulation that underlie the pathogenesis of endometriosis. The data are summarized in Table 1.

Statistical analysis revealed significant differences between patients with endometriosis and the control group in terms of the number of inflammatory, hematological, and immunological parameters. The concentration of C-reactive protein (CRP), an indicator of ongoing inflammation, was significantly higher in the study group (*p* < 0.0001), suggesting the presence of a chronic, systemic inflammatory process. Blood morphology revealed higher neutrophil counts (NEU) and significantly lower eosinophil counts (EOS) in patients with endometriosis (*p* < 0.0001), which may indicate changes in leukocyte composition typical of nonspecific inflammation. In terms of humoral immunity, a significantly reduced IgG concentration was observed, with simultaneous increases in IgM and IgA levels (*p* < 0.0001). This may indicate a different profile of the immune response associated with the presence of the disease.

Phenotypic analysis of mononuclear cells revealed a significantly lower percentage of CD45+, CD3+, CD4+, CD8+, and CD19+ cells in the patient population with endometriosis compared to the healthy women in the control group. At the same time, the percentage of NK cells was moderately but significantly higher (*p* = 0.042) in the study group.

**Figure 1:**
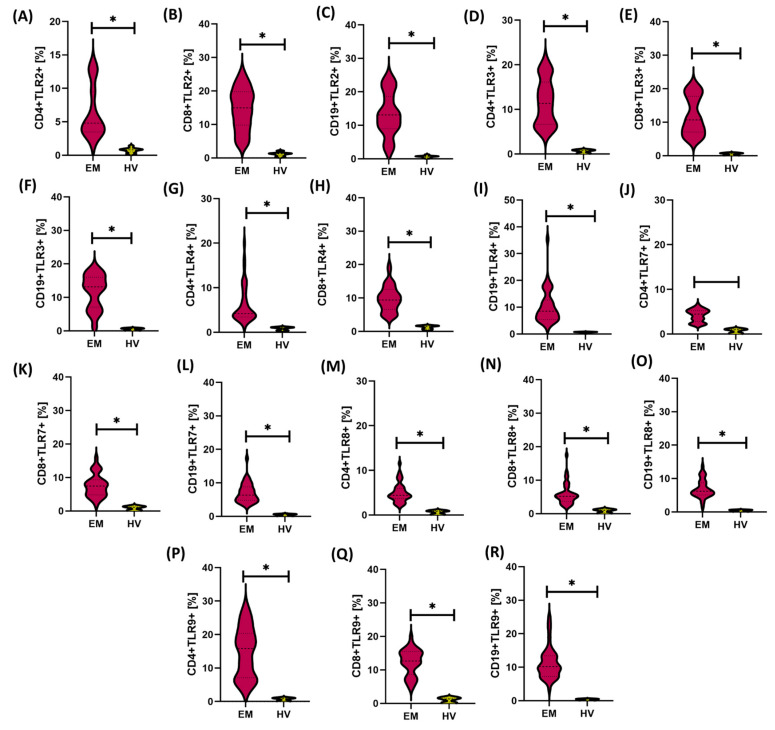
(**A**) Percentage of CD4+ T helper lymphocytes expressing TLR2 in patients with endometriosis (EM) and healthy volunteers (HV); (**B**) Percentage of CD8+ T helper lymphocytes expressing TLR2 in patients with endometriosis (EM) and healthy volunteers (HV); (**C**) Percentage of CD19+ T helper lymphocytes expressing TLR2 in patients with endometriosis (EM) and healthy volunteers (HV); (**D**) Percentage of CD4+ T helper lymphocytes expressing TLR3 in patients with endometriosis (EM) and healthy volunteers (HV); (**E**) Percentage of CD8+ T helper lymphocytes expressing TLR3 in patients with endometriosis (EM) and healthy volunteers (HV); (**F**) Percentage of CD19+ T helper lymphocytes expressing TLR3 in patients with endometriosis (EM) and healthy volunteers (HV); (**G**) Percentage of CD4+ T helper lymphocytes expressing TLR4 in patients with endometriosis (EM) and healthy volunteers (HV); (**H**) Percentage of CD8+ T helper lymphocytes expressing TLR4 in patients with endometriosis (EM) and healthy volunteers (HV); (**I**) Percentage of CD19+ T helper lymphocytes expressing TLR4 in patients with endometriosis (EM) and healthy volunteers (HV); (**J**) Percentage of CD4+ T helper lymphocytes expressing TLR7 in patients with endometriosis (EM) and healthy volunteers (HV); (**K**) Percentage of CD8+ T helper lymphocytes expressing TLR7 in patients with endometriosis (EM) and healthy volunteers (HV); (**L**) Percentage of CD19+ T helper lymphocytes expressing TLR7 in patients with endometriosis (EM) and healthy volunteers (HV); (**M**) Percentage of CD4+ T helper lymphocytes expressing TLR8 in patients with endometriosis (EM) and healthy volunteers (HV); (**N**) Percentage of CD8+ T helper lymphocytes expressing TLR8 in patients with endometriosis (EM) and healthy volunteers (HV); (**O**) Percentage of CD19+ T helper lymphocytes expressing TLR8 in patients with endometriosis (EM) and healthy volunteers (HV); (**P**) Percentage of CD4+ T helper lymphocytes expressing TLR9 in patients with endometriosis (EM) and healthy volunteers (HV); (**Q**) Percentage of CD8+ T helper lymphocytes expressing TLR9 in patients with endometriosis (EM) and healthy volunteers (HV); (**R**) Percentage of CD19+ T helper lymphocytes expressing TLR9 in patients with endometriosis (EM) and healthy volunteers (HV). Data are presented as violin plots, showing the median and distribution of values. In all analyzed subpopulations, a significantly higher percentage of cells positive for a given TLR was found in the group of patients with endometriosis compared to the control group (*p* < 0.05). The results indicate an increased expression profile of innate immune response receptors in patients with endometriosis. Statistically significant differences are indicated by an asterisk (*).

The next element of the analysis was the assessment of the expression of Toll-like receptors (TLR2, TLR3, TLR4, TLR7, TLR8, and TLR9) on the surface of T helper lymphocytes (CD4+), cytotoxic T lymphocytes (CD8+), and B lymphocytes (CD19+). In all analyzed subpopulations, a significantly higher percentage of cells expressing the studied TLRs was found in patients with endometriosis compared to the control group. Increased expression of these receptors may reflect dysregulation of immune regulatory mechanisms and the chronic inflammatory state accompanying the disease. The results are summarized in Figure 1.

**Figure 2:**
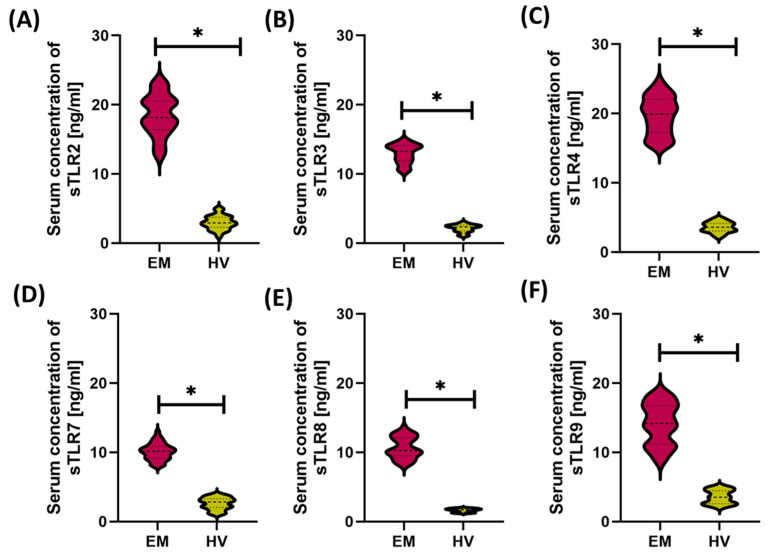
Concentrations of soluble forms of Toll-like receptors (sTLR2, sTLR3, sTLR4, sTLR7, sTLR8, sTLR9) in the serum of patients with endometriosis (EM) and healthy volunteers (HV). (**A**) Serum concentration of sTLR2 in EM and HV groups; (**B**) Serum concentration of sTLR3 in EM and HV groups; (**C**) Serum concentration of sTLR4 in EM and HV groups; (**D**) Serum concentration of sTLR7 in EM and HV groups; (**E**) Serum concentration of sTLR8 in EM and HV groups; (**F**) Serum concentration of sTLR9 in EM and HV groups. In each of the analyzed receptor groups, significantly higher sTLR concentrations were observed in the serum of women with endometriosis compared to the control group (*p* < 0.05). Data are presented as violin plots, showing the median and distribution of values. Statistically significant differences are indicated by an asterisk (*).

**Figure 3:**
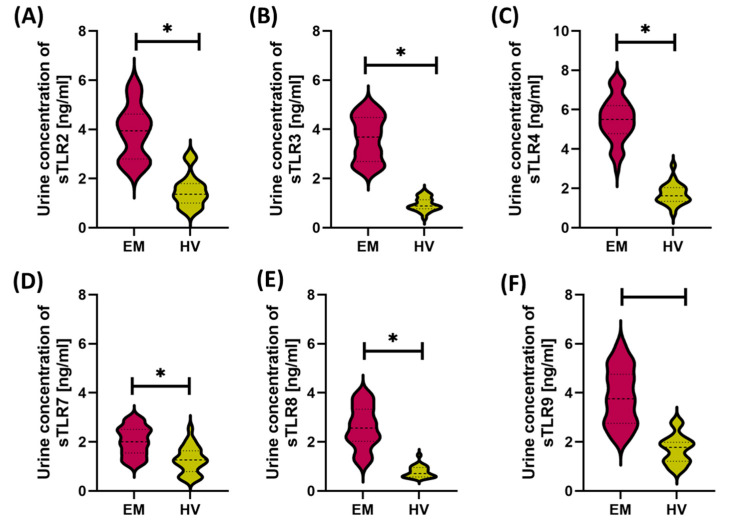
Concentrations of soluble forms of Toll-like receptors (sTLR2, sTLR3, sTLR4, sTLR7, sTLR8, sTLR9) in the urine of patients with endometriosis (EM) and healthy volunteers (HV). (**A**) Urine concentration of sTLR2 in EM and HV groups; (**B**) Urine concentration of sTLR3 in EM and HV groups; (**C**) Urine concentration of sTLR4 in EM and HV groups; (**D**) Urine concentration of sTLR7 in EM and HV groups; (**E**) Urine concentration of sTLR8 in EM and HV groups; (**F**) Urine concentration of sTLR9 in EM and HV groups. In all cases, significantly higher concentrations of sTLRs were observed in the urine of patients with endometriosis compared to the control group (*p* < 0.05), indicating the potential involvement of local inflammatory mechanisms in disease progression. Statistically significant differences are indicated by an asterisk (*).

Analysis of the concentrations of soluble forms of Toll-like receptors (sTLR2, sTLR3, sTLR4, sTLR7, sTLR8, and sTLR9) in the serum and urine of patients with endometriosis and healthy women revealed significant differences both between the studied groups and between the analyzed body fluids. In both groups—patients with endometriosis (EM) and healthy volunteers (HV)—sTLR concentrations in serum were significantly higher than in urine (Figure 2 and Figure 3). The highest serum values in the EM group were noted for sTLR2, sTLR3, and sTLR4. Despite lower concentrations in urine, the levels of these same receptors were also significantly increased compared to the control group, suggesting their potential importance at the local level as well. sTLR7, sTLR8, and sTLR9 showed a similar pattern—significantly higher concentrations in serum than in urine—and these values were increased in patients with endometriosis compared to healthy individuals. An apparent difference was observed for sTLR8 and sTLR9, whose significantly increased serum levels may indicate their dominant participation in the systemic immune response and chronic inflammatory process associated with endometriosis.

### 3.2. Comparison of Immunological and Biochemical Parameters in Patients with Different Forms of Endometriosis: Peritoneal, Ovarian, Deeply Infiltrating, and Cesarean Section Scar

Endometriosis exhibits significant morphological and anatomical heterogeneity, which is reflected in the clinical classification of the disease into several main types, differing in location, characteristics of the lesions, and their impact on bodily functions. In the next stage of the study, a comparison of selected immunological and biochemical parameters was conducted between patients with the above-mentioned forms of the disease and the HV group (Table 2).

The analysis aimed to identify potential differences in systemic inflammatory markers, immunoglobulin profiles, and immune cell phenotypes, which could reflect varying immune activity and the severity of pathological processes depending on the form of the disease.

Detailed results of the comparative analyses are presented in Table S2 and Figure S4 of the Supplementary Materials.

**Figure 4:**
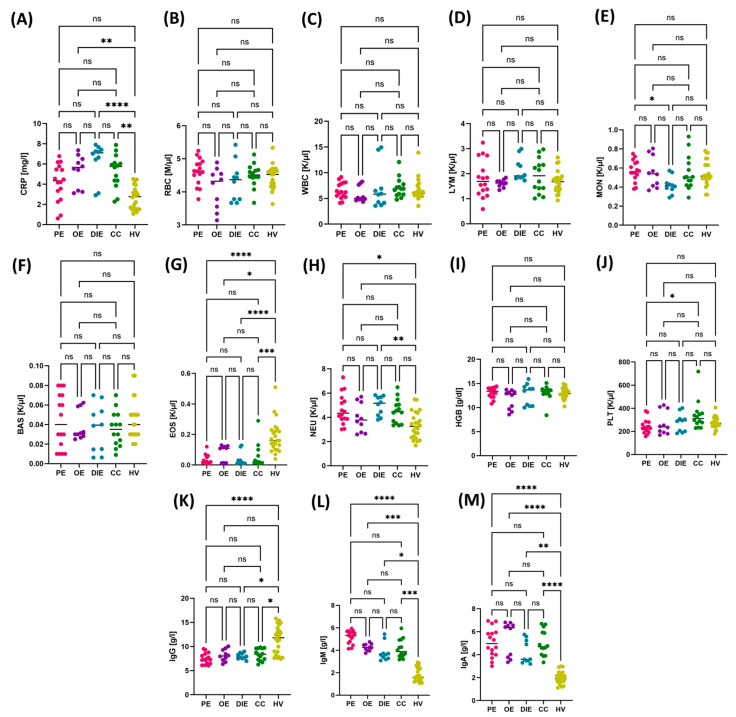
Comparison of selected hematological and immunological parameters between patients with different forms of endometriosis—peritoneal (PE), ovarian (OE), deeply infiltrating (DIE), cesarean section scar (CC)—and healthy volunteers (HV). Statistical significance values are marked as follows: *p* ≤ 0.05 (*), *p* ≤ 0.01 (**), *p* ≤ 0.001 (***), *p* ≤ 0.0001 (****), ns—no statistical significance. (**A**) Comparison concentration of CRP between patients with PE, OE, DIE, CC, HV; (**B**) Comparison concentration of RBC between patients with PE, OE, DIE, CC, HV; (**C**) Comparison concentration of WBC between patients with PE, OE, DIE, CC, HV; (**D**) Comparison concentration of LYM between patients with PE, OE, DIE, CC, HV; (**E**) Comparison concentration of MON between patients with PE, OE, DIE, CC, HV; (**F**) Comparison concentration of BAS between patients with PE, OE, DIE, CC, HV; (**G**) Comparison concentration of EOS between patients with PE, OE, DIE, CC, HV; (**H**) Comparison concentration of NEU between patients with PE, OE, DIE, CC, HV; (**I**) Comparison concentration of HGB between patients with PE, OE, DIE, CC, HV; (**J**) Comparison concentration of PLT between patients with PE, OE, DIE, CC, HV; (**K**) Comparison concentration of IgG between patients with PE, OE, DIE, CC, HV; (**L**) Comparison concentration of IgM between patients with PE, OE, DIE, CC, HV; (**M**) Comparison concentration of IgA between patients with PE, OE, DIE, CC, HV.The next part of the analysis focused on assessing the concentrations of soluble forms of TLRs (sTLR2, sTLR3, sTLR4, sTLR7, sTLR8, and sTLR9) in two types of biological material—serum and urine—from patients with endometriosis. The aim was to determine whether there were differences in the concentration patterns of the analyzed receptors between the individual subtypes and in comparison to the HV group. The numerical data are included in Table S4 of the Supplementary Materials. Analysis of the results concerning serum concentrations showed that all assessed sTLRs were significantly increased in patients with endometriosis, regardless of its form, compared to the control group. At the same time, no significant differences were noted in serum sTLR concentrations between the studied endometriosis subtypes, which may suggest that the increase in these markers is systemic and independent of the location of ectopic endometrial changes (Figure 5).

In the assessment of individual hematological parameters between different forms of endometriosis—peritoneal (PE), ovarian (OE), deeply infiltrating (DIE), and cesarean section scar (CC)—no statistically significant differences were found in the number of erythrocytes (RBC), total leukocytes (WBC), lymphocytes (LYM), basophils (BAS), or hemoglobin concentration (HGB), both in comparisons between the endometriosis subtypes and in relation to the control group (HV) (Figure 4B–D,F,I).

The variability was concerned primarily with inflammatory markers and selected immunological parameters. Significantly higher concentrations of CRP were observed in patients with OE, DIE, and CC compared to healthy women, which confirms the intense nature of the inflammatory reaction in these forms of the disease (Figure 4A). Additionally, monocytes (MON) showed significantly higher values in the PE group compared to the DIE group (Figure 4E).

Granulocyte analysis showed that the eosinophil level (EOS) was significantly lower in all endometriosis subtypes compared to the HV group. In turn, the number of neutrophils (NEU) was increased in the PE and CC groups compared to the HV group, which may reflect a nonspecific inflammatory component (Figure 4G,H).

Regarding platelet count (PLT), significantly higher values were noted in patients with endometriosis in the CC group compared to the PE group, which may suggest the presence of local regenerative and inflammatory processes (Figure 4J).

In terms of humoral response, significantly reduced immunoglobulin G (IgG) levels were found in patients with PE, DIE, and CC compared to the HV group. However, the levels of immunoglobulins M and A (IgM and IgA) were significantly increased in all analyzed endometriosis subtypes compared to healthy women, which may indicate the activation of the nonspecific immune response and the mucosal component in the pathogenesis of the disease (Figure 4K–M).

In all analyzed lymphocyte subpopulations, patients with endometriosis demonstrated a significantly higher percentage of TLR-expressing cells compared to healthy volunteers (HV), indicating enhanced immune activation. Among CD4+ T cells, a markedly increased expression of all studied TLRs was observed in the endometriosis groups (*p* < 0.0001 for most comparisons), with the highest levels noted for TLR3 and TLR9. Notably, in the case of TLR4 expression, significant differences were also found between specific disease subtypes—particularly between cesarean section scar endometriosis (CC) and deeply infiltrating endometriosis (DIE)—suggesting distinct patterns of immunopathogenesis (Supplementary Materials, Figure S11C and Table S3). A similar pattern was observed in cytotoxic CD8+ T lymphocytes, where all subtypes of endometriosis were associated with a significantly elevated percentage of CD8^+^+LR^+^ cells compared to the HV group. The only statistically significant difference between disease subtypes within this population was noted for the CD8+TLR4+subset, which was more prominent in patients with CC compared to DIE (Supplementary Materials, Figure S12C and Table S3). In the case of B cells (CD19+), significantly higher percentages of cells expressing TLR2–TLR4 and TLR7–TLR9 were observed in all endometriosis subtypes compared to the HV group (Supplementary Materials, Figure S13). However, despite observable differences in medians between disease subtypes, no statistically significant intergroup differences were found within the CD19+TLR+ population (Supplementary Materials, Table S3).

**Figure 5:**
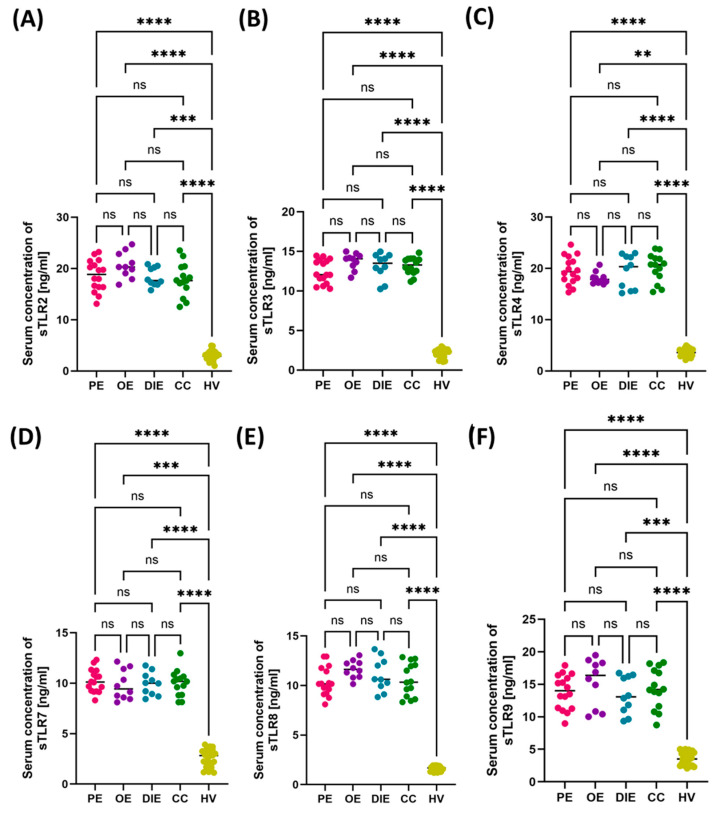
Concentrations of soluble forms of Toll-like receptors (sTLR2–sTLR8) in the serum of patients with different subtypes of endometriosis—peritoneal (PE), ovarian (OE), deeply infiltrating (DIE), and cesarean section scar (CC)—compared with healthy volunteers (HV). Concentrations of (**A**) sTLR2, (**B**) sTLR3, (**C**) sTLR4, (**D**) sTLR7, (**E**) sTLR8, and (**F**) sTLR9 are presented. Statistical significance values are marked as follows: *p* ≤ 0.01 (**), *p* ≤ 0.001 (***), *p* ≤ 0.0001 (****), ns—no statistical significance.

**Figure 6:**
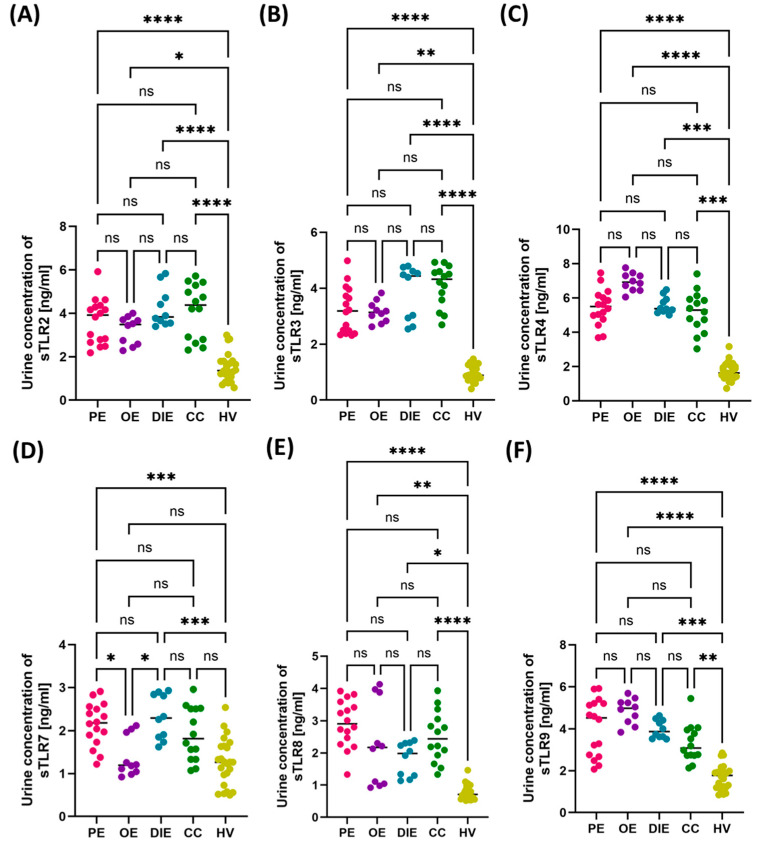
Concentrations of soluble forms of Toll-like receptors (sTLR2–sTLR9) in the urine of patients with different forms of endometriosis—peritoneal (PE), ovarian (OE), deeply infiltrating (DIE), and cesarean section scar (CC)—compared with healthy volunteers (HV). Concentrations of (**A**) sTLR2, (**B**) sTLR3, (**C**) sTLR4, (**D**) sTLR7, (**E**) sTLR8, and (**F**) sTLR9 are presented. Statistical significance values are marked as follows: *p* ≤ 0.05 (*), *p* s≤ 0.01 (**), *p* ≤ 0.001 (***), *p* ≤ 0.0001 (****), ns—no statistical significance.

In the analysis of the concentrations of soluble forms of Toll-like receptors (sTLRs) in urine samples from patients with endometriosis, a general trend similar to that observed in serum was observed. In the case of sTLR2, sTLR3, sTLR4, sTLR8, and sTLR9, the concentration values were significantly increased in all subtypes of endometriosis compared to healthy volunteers, with no significant differences between the subtypes of the disease (Figure 6A–C,E,F). The obtained results indicate that the increased excretion of these soluble receptors into urine may be a general phenomenon accompanying endometriosis, regardless of the location of the disease foci.

However, a different pattern was observed for sTLR7. In this case, statistical analysis revealed significant differences not only between the patient group and the HV group but also between selected subtypes of endometriosis. Urinary sTLR7 levels were significantly higher in PE and DIE patients compared to the HV group. Additionally, significant differences were observed between PE and OE, as well as between DIE and OE, which may suggest a different mechanism of TLR7 involvement in individual clinical forms of the disease (Figure 6D).

### 3.3. Diagnostic Value of TLR Expression and Their Soluble Forms in Endometriosis—ROC Curve Analysis

**Figure 7:**
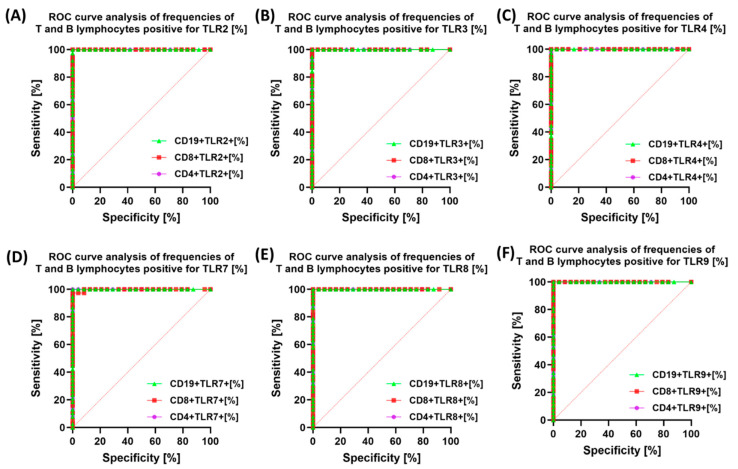
ROC curves illustrating the discriminatory ability of the percentage expression of the tested Toll-like receptors (TLRs) on selected subpopulations of immune system cells (**A**–**F**) in patients with endometriosis compared with healthy volunteers.

In the next stage of our research, we decided to use ROC curves to check the ability of the tested TLRs to differentiate the study group (e.g., patients with endometriosis) from the HV group based on parameters such as sensitivity and specificity. The obtained results are presented in Figure 7. All analyzed TLRs (TLR2, TLR3, TLR4, TLR7, TLR8, and TLR9) exhibited high sensitivity and specificity in the diagnosis of endometriosis, suggesting that their expression on CD4+, CD8+, and CD19+ lymphocytes may serve as a potential marker for differentiating patients with endometriosis from healthy women.

**Figure 8:**
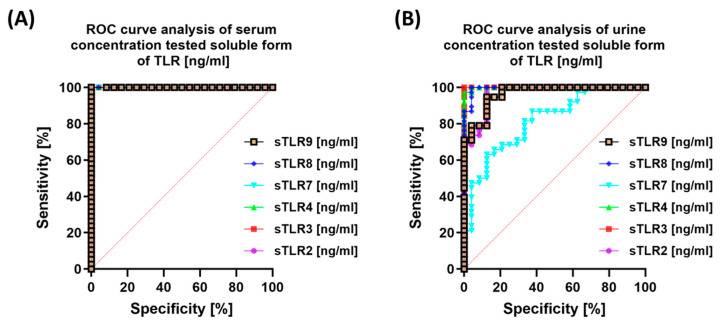
ROC curves showing the diagnostic value of soluble forms of Toll-like receptors (sTLRs) in serum (**A**) and urine (**B**) from patients with endometriosis in comparison to healthy volunteers.

We observed similar trends when analyzing the serum concentrations of the studied sTLRs (Figure 8A). In urine, sTLR3 was the most sensitive, while sTLR7 was the least sensitive and specific (Figure 8B).

The same analysis was performed for individual subtypes of endometriosis, and the results are presented in Table S5 of the Supplementary Materials. The highest diagnostic value (AUC = 1.0) was demonstrated by CD4 + TLR2, CD4 + TLR3, CD4 + TLR4, CD4 + TLR9, CD8 + TLR2, CD8 + TLR3, CD8 + TLR8, CD19 + TLR2, CD19 + TLR3, and CD19 + TLR9 in the comparisons between PE and HV, as well as OE and HV. High AUC values for these biomarkers confirm their efficacy in distinguishing between the groups and suggest their potential use in diagnosing endometriosis. Similarly, the expression of the same markers in the comparisons of DIE vs. HV and CC vs. HV indicates their usefulness in identifying patients with a more advanced form of the disease. In the case of differentiation between endometriosis phenotypes, AUC values ≥ 0.85 were obtained for CD8 + TLR3, CD4 + TLR4, and CD19 + TLR3 in the comparisons of PE vs. CC and DIE vs. CC, suggesting their importance in distinguishing these disease subtypes. Additionally, the expression of CD19 + TLR9 in the comparisons of PE vs. CC (AUC = 0.86) and OE vs. CC (AUC = 0.92) may indicate that TLR9 on B lymphocytes is mainly associated with more advanced forms of endometriosis. Several molecules showed moderate diagnostic value (AUC ranging from 0.7 to 0.85), especially in differentiating between endometriosis phenotypes. Among them, CD4 + TLR2 and CD8 + TLR2 in the comparison of PE vs. DIE (AUC = 0.80) suggest that TLR2 expression increases significantly in DIE compared to PE. Furthermore, CD4 + TLR4 in the comparison of PE vs. CC (AUC = 0.86) confirms the role of this receptor in more advanced forms of endometriosis. High AUC values were also noted for CD8+-TLR8 in the comparisons of PE vs. CC (AUC = 0.88) and OE vs. CC (AUC = 0.88), which may indicate activation of innate immunity in CC. CD19 + TLR3 also demonstrated significant diagnostic value in the comparisons of PE versus CC (AUC = 0.93) and OE versus CC (AUC = 0.95).

Analysis of the diagnostic capacity of sTLR in the serum of patients with endometriosis showed that the highest efficacy in differentiating these patients from healthy women (AUC = 1.0) was achieved by sTLR2, sTLR3, sTLR4, sTLR7, sTLR8, and sTLR9 concentrations in the comparisons of PE vs. HV, OE vs. HV, DIE vs. HV, and CC vs. HV (Supplementary Materials, Table S2). In the context of differentiating endometriosis phenotypes, moderate diagnostic value was demonstrated by sTLR2 (AUC = 0.833) in the comparison of OE vs. CC; sTLR3 (AUC = 0.813) in the comparison of PE vs. OE; and sTLR8 (AUC = 0.813) in the comparison of PE vs. OE. In the assessment of the usefulness for differentiating endometriosis phenotypes, moderate diagnostic value was obtained for sTLR2 in urine (AUC = 0.80) in the comparison of OE vs. DIE; sTLR3 in urine (AUC = 0.857) in the comparison of OE vs. CC; sTLR4 in urine (AUC = 0.922) in the comparisons of PE vs. OE and OE vs. DIE (AUC = 0.933) or OE vs. CC (AUC = 0.929); sTLR7 in urine (AUC = 0.896) in the comparisons of PE vs. OE and OE vs. DIE (AUC = 0.867); sTLR8 in urine (AUC = 0.875) in the comparison of PE vs. DIE; and sTLR9 in urine (AUC = 0.933 and 0.929) in the comparisons of OE vs. DIE and OE vs. CC, respectively.

## 4. Discussion

Endometriosis is a multifactorial chronic disease characterized by the ectopic presence of endometrial glands and stroma outside the uterine cavity. Its development and progression are closely linked to a dysregulated immune response [37]. Clinically, endometriosis exhibits significant morphological and anatomical heterogeneity, which underlies its classification into several main types, differing in location, characteristics of lesions, and their effect on organ function. The most commonly distinguished subtypes include the following:

- Peritoneal endometriosis (PE): Involves superficial lesions on the serosa of the abdominal and pelvic cavities. These lesions are typically multifocal, small, and vary in color (red, black, or white), often accompanied by local fibrosis and inflammatory infiltration. PE is most frequently diagnosed during laparoscopy and commonly manifests as chronic pelvic pain.
- Ovarian endometriosis (OE): Characterized by the presence of endometriotic ovarian cysts (so-called “chocolate cysts”) filled with thick, hemorrhagic content. These lesions contribute to structural damage to ovarian tissue, reduced ovarian reserve, and decreased fertility.
- Deeply infiltrating endometriosis (DIE): The most severe form, defined by lesions penetrating more than 5 mm into extraperitoneal tissues. DIE may affect the sacrotuberous ligaments, rectovaginal septum, bladder wall, intestines, and, rarely, the diaphragm or lungs. This form is typically associated with severe pelvic pain, dyspareunia, gastrointestinal symptoms, and infertility.
- Cesarean section scar endometriosis (CC): A less frequent form, occurring in the subcutaneous tissue or muscle layers of the anterior abdominal wall, usually in the region of surgical scars following cesarean delivery. It typically presents as cyclic pain localized to the scar area that intensifies during menstruation.

Considering these subtypes is of high diagnostic and therapeutic relevance, as each form may differ in clinical presentation, immune response profile, rate of progression, and treatment efficacy. These variations are also believed to reflect distinct underlying pathophysiological mechanisms and immune dysregulations specific to the location and type of ectopic endometrial lesions.

Among the key players in this immune dysfunction are TLRs, which serve critical roles in innate immunity by recognizing PAMPs and DAMPs and by orchestrating downstream inflammatory signaling pathways [38,39].

In particular, TLR4 has garnered considerable attention for its significant involvement in the pathophysiology of endometriosis. Aberrant expression and polymorphisms in the *TLR4* gene may contribute to the initiation of ectopic lesions and the severity of clinical symptoms. Activation of TLR4 by lipopolysaccharides (LPS) of bacterial origin triggers the release of pro-inflammatory cytokines such as interleukin-6 (IL-6) and tumor necrosis factor-alpha (TNF-α), thus creating a chronic inflammatory microenvironment that promotes the persistence and proliferation of endometrial lesions [40,41,42]. The detection of bacteria such as *Escherichia coli* in menstrual blood further supports the concept that both exogenous and endogenous ligands can activate TLR4 pathways, thereby exacerbating the inflammatory responses associated with endometriosis [43,44].

The interaction of endometrial cells with LPS and the subsequent activation of TLR4 not only intensifies local inflammation but may also enhance ectopic tissue survival and expansion. Notably, specific polymorphisms, such as *TLR4* A896G (D299G), have been implicated in reduced receptor responsiveness, potentially impairing innate immune defenses and fostering an environment conducive to lesion implantation and immune evasion [45,46]. Such impaired immune surveillance facilitates the adhesion of endometrial cells to peritoneal surfaces and contributes to lesion establishment [20]. Altered TLR4 expression has also been documented in various immune cell populations within the peritoneal cavity of women with endometriosis, highlighting immune dysfunctions that support chronic inflammation [13,15].

Beyond TLR4, TLR2 also appears to play a significant role in the pathogenesis of endometriosis. Elevated TLR2 expression has been associated with advanced disease stages, suggesting its utility as a potential biomarker [47]. The regulation of TLR2 is modulated by local cytokines and inflammatory mediators, underscoring the complexity of the endometriotic microenvironment [20,48]. Moreover, TLR signaling in peripheral immune cells may modulate pain perception in endometriosis through inflammatory mediators, revealing a mechanistic link between immune dysregulation and symptomatology [49,50].

Given the growing body of evidence implicating the immune system in the progression of endometriosis, comprehensive profiling of lymphocyte subpopulations has become increasingly relevant. Regulatory T cells (Tregs), which express TLRs, may exhibit enhanced immunosuppressive capabilities that inhibit effector responses, thus facilitating the survival of ectopic endometrial tissue [51,52]. Concurrently, there is a shift in CD4+ T cell polarization toward the Th2 phenotype, which supports the production of anti-inflammatory cytokines and endometrial cell persistence at ectopic sites [53,54]. Elevated B cell activity and numbers have also been linked to disease progression [55,56]. In contrast, CD8+ T cells and NK cells exhibit impaired cytotoxic function, which is partially attributed to the increased expression of inhibitory receptors [57,58,59,60,61,62,63]. These immune alterations, combined with the heightened secretion of pro-inflammatory cytokines such as IL-6, foster a self-perpetuating inflammatory state in the peritoneal cavity [64,65,66].

Our investigations revealed significant alterations in the distribution of immune cell subpopulations in women with endometriosis compared to healthy volunteers. We observed markedly higher expression of TLR2, TLR3, TLR4, TLR7, TLR8, and TLR9 on CD4+, CD8+, and CD19+ lymphocytes in the endometriosis group, indicating their active participation in modulating immune responses in the disease setting. Importantly, we also report—for the first time—significantly elevated levels of soluble TLRs (sTLR2, sTLR3, sTLR4, sTLR7, sTLR8, and sTLR9) in both the serum and urine from patients with endometriosis. These findings suggest a dual role for sTLRs in systemic and local immunoregulation, potentially through receptor-shedding or scavenging mechanisms that modulate TLR signaling.

sTLRs can be detected in various biological fluids, including serum and urine; however, their concentrations in these compartments may differ significantly due to several physiological and biochemical factors [67,68,69]. The generally lower urinary concentrations of sTLRs compared to serum may be attributed to the selective permeability of the glomerular filtration barrier, which limits the passage of high-molecular-weight or structurally complex proteins. Additionally, differential pharmacokinetics—such as the more rapid clearance of sTLR4 relative to sTLR2—may also influence their bioavailability in peripheral fluids. A study by Oever et al. demonstrated that despite equivalent in vitro release of sTLR2 and sTLR4 by immune cells, plasma concentrations of sTLR2 were consistently higher, likely due to accelerated elimination of sTLR4 [70]. Following LPS administration in humans, sTLR4 reached its peak concentration approximately 2 h post-exposure, while sTLR2 peaked around 4 h, further confirming differences in clearance kinetics [70].

Moreover, the biochemical composition of urine—including the presence of proteases, binding proteins, and variable pH—may compromise the stability and detectability of sTLRs [71]. Notably, differences in urinary sTLR concentrations among various clinical forms of endometriosis (OE, PE, DIE, CC) may reflect distinct local inflammatory and immunological microenvironments. These phenotypes not only differ in lesion location but also tissue invasiveness, inflammatory intensity, and oxidative stress, all of which may influence TLR expression and shedding [72,73,74]. Additionally, variations in local transcriptional control via NF-κB or STAT pathways, as well as the diversity of cellular sources of sTLRs—including epithelial, stromal, and immune cells—may underlie phenotype-specific differences in urinary sTLR profiles [75,76,77].

Taken together, this multifaceted regulatory landscape must be considered when interpreting sTLR data and evaluating their suitability as non-invasive biomarkers for endometriosis.

As supported by the literature, deregulated TLR signaling represents a crucial aspect of endometriosis pathophysiology, involving both innate and adaptive immune dysfunction. Future research exploring TLR signaling cascades, gene polymorphisms, and intercellular immune interactions will be crucial for elucidating the mechanisms underlying this disease and for developing novel diagnostic tools and therapeutic targets [10,12,78].

Despite the promising implications of our findings, several limitations should be acknowledged. First, the relatively small sample size restricts the generalizability of our conclusions and precludes more granular subgroup analyses (e.g., disease stage or lesion type). Second, while flow cytometry enabled quantification of TLR expression on lymphocytes, it did not permit functional assessments of receptor activity or intracellular signaling. This study also did not evaluate the interplay between lymphocytes and other immune cell types (e.g., macrophages, neutrophils), which may be critical contributors to the disease process. The cross-sectional nature of this study prevents conclusions about the temporal dynamics of TLR expression or its modulation by treatment. Longitudinal studies are needed to capture these changes over time. Furthermore, the lack of correlation between TLR expression and clinical variables (e.g., pain severity, treatment outcomes) limits the immediate clinical applicability of the findings. Future studies should include control groups with other inflammatory or gynecological conditions to enhance diagnostic specificity and refine the utility of TLR-related biomarkers.

## 5. Conclusions

In conclusion, the present study confirms the critical involvement of TLRs in the immunopathogenesis of endometriosis. The observed upregulation of TLR2, TLR3, TLR4, TLR7, TLR8, and TLR9 on peripheral lymphocyte subsets, alongside the elevated concentrations of their soluble forms in serum and urine, suggests a potential role for these receptors in orchestrating both local and systemic inflammatory responses associated with the disease.

The diagnostic utility of TLRs is further supported by the results of the ROC curve analyses, which demonstrate the high sensitivity and specificity of selected TLRs in distinguishing patients with endometriosis from healthy controls. Moreover, the ability of these markers to differentiate between disease phenotypes suggests their potential application in refining diagnostic stratification within endometriosis subtypes.

Despite these promising findings, several important considerations remain. Future research should encompass larger and more diverse patient cohorts to enhance the robustness and generalizability of the results. Additionally, mechanistic studies focusing on intracellular TLR signaling pathways and intercellular immune interactions will be crucial to better understand the functional implications of TLR dysregulation in endometriosis.

Longitudinal studies are particularly warranted to investigate the temporal dynamics of TLR expression in disease progression, symptom severity, and treatment response. Furthermore, the inclusion of disease-specific control groups—particularly those with other inflammatory or gynecological conditions—will be essential for establishing the specificity and clinical relevance of TLR-based biomarkers.

In the long term, these insights may inform the development of novel therapeutic strategies aimed at modulating TLR activity, thereby attenuating chronic inflammation and halting the progression of endometriotic lesions, ultimately improving clinical outcomes for affected patients.

## Figures and Tables

**Table 1:** Comparison of basic hematological parameters, inflammatory markers, and the percentage distribution of immune cell subpopulations between patients with endometriosis and healthy controls.

Parameters		EM (*n* = 50)		HV (*n* = 24)		*p*-Value
		Median	Q1–Q3	Median	Q1–Q3	
Hematological, inflammatory, and immunoglobulin parameters	RBC [M/µL]	4.52	4.10–4.71	4.53	4.18–4.64	0.850
	WBC [K/µL]	6.10	5.04–7.73	6.01	5.71–7.15	0.718
	LYM [K/µL]	1.79	1.43–2.29	1.68	1.39–1.83	0.180
	MON [K/µL]	0.52	0.42–0.60	0.52	0.49–0.63	0.626
	BAS [K/µL]	0.03	0.02–0.06	0.04	0.03–0.05	0.367
	EOS [K/µL]	0.02	0.01–0.07	0.16	0.11–0.23	<0.0001 *
	NEU [K/µL]	4.43	3.68–5.43	3.26	2.38–4.16	<0.0001 *
	HGB [g/dL]	13.15	12.20–13.85	12.90	12.50–13.80	0.895
	PLT [K/µL]	256.55	206.75–335.00	270.5	249.00–302.50	0.562
	CRP [mg/L]	5.61	3.51–6.60	2.76	1.54–3.32	<0.0001 *
	IgG [g/L]	7.84	7.20–8.75	11.81	8.53–13.55	<0,0001 *
	IgM [g/L]	4.28	3.71–5.10	1.59	1.38–2.33	<0,0001 *
	IgA [g/L]	4.70	3.76–5.94	1.95	1.68–2.36	<0,0001 *
Immunophenotypic characterization of lymphocyte subpopulations	CD45+ [%]	87.86	81.86–91.80	96.28	92.83–98.05	<0.0001 *
	CD3+ [%]	66.02	59.95–74.20	72.34	70.70–76.54	0.0012 *
	CD4+ [%]	41.84	27.20–51.37	47.49	44.77–49.84	0.0144 *
	CD8+ [%]	20.51	17.31–28.39	27.23	25.88–28.52	0.0021 *
	CD19+ [%]	11.27	10.17–14.35	12.70	11.82–13.69	0.032 *
	NK cells [%]	9.24	4.26–17.72	7.90	7.67–10.89	0.042 *

The following abbreviations are used: BAS—basophils; CD—cluster of differentiation (including CD3—T lymphocytes; CD4—T helper lymphocytes; CD8—cytotoxic T lymphocytes; CD19—B lymphocytes; CD45—total leukocytes); CRP—C-reactive protein; EOS—eosinophils; HGB—hemoglobin; IgA—immunoglobulin A; IgG—immunoglobulin G; IgM—immunoglobulin M; LYM—lymphocytes; MON—monocytes; NK cells—natural killer cells; NEU—neutrophils; PLT—platelets; RBC—red blood cells; and WBC—white blood cells. [%] Refers to the percentage of cells positive for the tested marker in the total population of analyzed cells, * statistically significant difference (*p* < 0.05).

**Table 2:** Demographic characteristics of patients with different subtypes of endometriosis and the control group.

Group	*n*	Median Age (Years)	Age Range (Years)
Peritoneal endometriosis (PE)	16	33	26–46
Ovarian endometriosis (OE)	10	35.5	26–47
Deeply infiltrating endometriosis (DIE)	10	36.5	27–47
Cesarean section scar endometriosis (CC)	14	34	24–42
HV	24	33	18–57

## Data Availability

The data presented in this publication are available from the first author upon written request.

## Supplementary Materials

The following supporting information can be downloaded at https://www.mdpi.com/article/10.3390/cells14161273/s1, Figure S1: Gating strategy for lymphocyte subpopulations. (A) Forward scatter (FSC-A) versus side scatter (SSC-A) plot for initial lymphocyte gating. (B) CD45 versus SSC-A plot to define leukocytes. (C) CD3 vs CD4 plot for CD4+ T cells, (violet) (D) CD3 vs CD8 plot for CD8+ T cells, (blue) (E) CD3 vs CD19 plot for B cells (orange), (F) CD3 vs CD56+CD16 plot to define natural killer (NK) cells (green), characterized by CD3^−^/CD56+CD16+ phenotype. Figure S2: The figure shows the analysis of TLR2 and TLR4 expression on CD4+ T cells using flow cytometry, including a complete gating strategy and quality control of the assays. Panel (A) illustrates the CD4+ T cell population selected based on the simultaneous expression of the CD3 and CD4 surface markers. Fluorescence minus one (FMO) controls (shown in panels (B) and (D) were used to correctly define the fluorescence threshold and define the boundaries of positive expression; FMO controls for the PE dye (for TLR2) and the APC dye (for TLR4) distinguish the specific signal from autofluorescence and background. Panel (C) shows the level of TLR4 expression (APC dye) within the selected CD4+ cell population, while panel (E) shows the expression of TLR2 (PE dye) in the same population. The use of two independent FMO controls for different fluorescence channels allowed for a reliable assessment of the presence of TLRs on CD4+ T lymphocytes, minimizing the risk of misinterpretation of results resulting from overlapping fluorescence spectra or inhomogeneous background. Figure S3: The figure shows the analysis of TLR7 and TLR8 expression on CD4+ T lymphocytes using flow cytometry and fluorescence signal quality control. Panel (A) shows the gating of the CD3+CD4+ lymphocyte population, which is the starting point for further analyses. Panels (B) and (D) show FMO (fluorescence minus one) controls, respectively for the PE (TLR7) and APC (TLR8) dyes, which allow for precise determination of the positive fluorescence limit and elimination of errors resulting from autofluorescence and background. Panel (C) shows the level of TLR7 expression, while panel (E) shows TLR8 expression on the surface of CD4+ lymphocytes. The use of two independent FMO controls adapted to a specific fluorescence channel allowed for precise and reliable determination of the percentage of cells expressing the analyzed Toll-like receptors, increasing the accuracy and reproducibility of the obtained results. Figure S4: The figure shows the analysis of TLR3 and TLR9 expression on CD4+ T cells by flow cytometry using FMO control. Panel (A) shows the gating of the CD3+CD4+ subpopulation, which served as a starting point for further analysis of Toll-like receptor expression. Panels (B) and (D) show FMO (fluorescence minus one) controls for TLR3 (PE) and TLR9 (APC) detection channels, respectively, which allow precise determination of fluorescence signal positivity thresholds by eliminating the influence of autofluorescence and nonspecific background. Panel (C) shows the detected expression of TLR3, while panel (E) shows the expression of TLR9 on the surface of CD4+ T cells. The use of separate FMO controls for each fluorescence channel allowed for reliable and reproducible determination of the percentage of cells positive for a given receptor, increasing the accuracy of the analysis and the correct interpretation of results. Figure S5: The figure shows the analysis of TLR2 and TLR4 receptor expression on CD8+ T cells using flow cytometry, including FMO control. Panel (A) shows gating of CD3+CD8+ cells, which were the starting population for further analysis of Toll-like receptor expression. Panels (B) and (D), respectively, show FMO controls for TLR4 (APC) and TLR2 (PE) fluorescence channels, used to determine signal positivity thresholds by excluding nonspecific background and autofluorescence. Panel (C) shows the detected TLR4 expression, while panel (E) shows TLR2 expression on CD8+ cells. The use of FMO control allows for precise and reproducible determination of fluorescence gate boundaries for each receptor, which is crucial for reliable assessment of their presence on the analyzed effector cells of the immune system. Figure S6: The figure shows the analysis of TLR7 and TLR8 expression on CD8+ T cells using flow cytometry and the appropriate FMO controls. Panel (A) shows the gating of the CD3+CD8+ cell population, which was the basis for further analysis. Panels (B) and (D) show FMO controls for the appropriate fluorescence channels, allowing precise determination of TLR7 and TLR8 positivity thresholds by eliminating background signal and autofluorescence. Panel (C) shows the detected TLR7 expression, while panel (E) shows TLR8 expression on CD8+ T cells. The use of FMO controls allows for a reliable determination of which cells actually express the tested receptors, thus ensuring the accuracy and reproducibility of the flow cytometric analysis. Figure S7: The figure shows the analysis of TLR3 and TLR9 expression on CD8+ T cells using flow cytometry. Panel (A) shows the CD3+CD8+ cell population that was isolated for further analysis. Panels (B) and (D) show the FMO (fluorescence minus one) controls for TLR3 and TLR9 channels, respectively, which allow for determining the correct threshold of positivity by eliminating background signal and autofluorescence. Panel (C) shows TLR3 receptor expression, and panel (E) shows TLR9 receptor expression in CD8+ cells. The use of two independent FMO controls for each detection channel allows for precise determination of the range of positivity and ensures the reliability and reproducibility of the analysis of Toll-like receptor expression on cytotoxic lymphocytes. Figure S8: The figure shows the analysis of TLR2 and TLR4 expression on B cells (CD19+) using flow cytometry. Panel (A) shows gating of CD3^−^CD19+ lymphocytes, which were isolated for further analysis. In panels (B) and (D) are included appropriate FMO (fluorescence minus one) controls, allowing for determining the borderline of signal positivity for TLR2 and TLR4, taking into account autofluorescence and background. Panel (C) shows TLR4 expression, and panel (E) shows TLR2 expression within selected B lymphocytes. The use of two independent FMO controls allows for reliable and precise analysis of the expression level of the tested Toll-like receptors, eliminating the risk of false positive results. Figure S9: Na przedstawionej rycinie zaprezentowano analizę ekspresji receptorów TLR7 i TLR8 na limfocytach B (CD19+) metodą cytometrii przepływowej. W panelu (A) pokazano bramkowanie komórek CD3^−^CD19+, które zostały wyizolowane do dalszej analizy ekspresji TLR. Panele (B) i (D) zawierają odpowiednie kontrole FMO (fluorescence minus one) umożliwiające precyzyjne ustalenie progów pozytywności fluorescencji dla TLR7 i TLR8, z uwzględnieniem autofluorescencji i sygnału tła. Panel (C) przedstawia poziom ekspresji TLR7, natomiast panel (E) dotyczy ekspresji TLR8 na limfocytach B. Zastosowanie indywidualnych kontroli FMO dla każdego fluorochromu zapewnia wiarygodność uzyskanych wyników, pozwalając na rzetelną ocenę obecności receptorów TLR7 i TLR8 na komórkach CD19+. Figure S10: The figure shows the analysis of TLR3 and TLR9 expression on the surface of B lymphocytes (CD19+) using flow cytometry. The middle panel (A) shows the gating of the CD3^−^CD19+ cell population, which was selected for further analysis. Panels (B) and (D) contain FMO (fluorescence minus one) controls, which allow for precise determination of fluorescence thresholds for the appropriate markers, eliminating the influence of autofluorescence and enabling correct setting of gates for detection of the specific signal. Panel (C) shows the level of TLR3 receptor expression, while panel (E) shows the level of TLR9 receptor expression in the selected CD19+ cell population. The use of appropriate controls and uniform analysis conditions allows for a reliable assessment of the presence of the analyzed receptors in the B lymphocyte subpopulation. Figure S11: Percentage of T helper lymphocytes (CD4+) expressing selected Toll-like receptors: TLR2 (A), TLR3 (B), TLR4 (C), TLR7 (D), TLR8 (E) and TLR9 (F) in individual subtypes of endometriosis—peritoneal (PE), ovarian (OE), deeply infiltrating (DIE), in the cesarean section scar (CC)—and in healthy women (HV). Statistical significance values were marked as follows: *p* ≤ 0.05 (*), *p* ≤ 0.01 (**), *p* ≤ 0.001 (****), *p* ≤ 0.0001 (****), ns—no statistical significance. Figure S12: Percentage of cytotoxic T lymphocytes (CD8+) expressing selected Toll-like receptors: TLR2 (A), TLR3 (B), TLR4 (C), TLR7 (D), TLR8 (E) and TLR9 (F) in patients with various forms of endometriosis—peritoneal (PE), ovarian (OE), deeply infiltrating (DIE), endometriosis in the cesarean section scar (CC)—compared to the group of healthy women (HV). Statistical significance values were marked as follows: *p* ≤ 0.05 (*), *p* ≤ 0.01 (**), *p* ≤ 0.001 (****), *p* ≤ 0.0001 (****), ns—no statistical significance. Figure S13: Percentage of B lymphocytes (CD19+) expressing selected Toll-like receptors: TLR2 (A), TLR3 (B), TLR4 (C), TLR7 (D), TLR8 (E) and TLR9 (F) in patients with various forms of endometriosis—peritoneal (PE), ovarian (OE), deeply infiltrating (DIE), endometriosis in the cesarean section scar (CC)—compared to the group of healthy women (HV). Statistical significance values were marked as follows: *p* ≤ 0.05 (*), *p* ≤ 0.01 (**), *p* ≤ 0.001 (****), *p* ≤ 0.0001 (****), ns—no statistical significance. Table S1: List of conjugated monoclonal antibodies used for flow cytometric analysis, including fluorochrome conjugates, clones, manufacturers, and catalog numbers. Table S2: Comparison of selected hematological (CRP, RBC, WBC, LYM, MON, BAS, EOS, NEU, HGB, PLT), immunological (IgG, IgM, IgA) parameters, and immune cell subpopulations (CD45+, CD3+, CD4+, CD8+, CD19+) between patients with different forms of endometriosis: peritoneal (PE; group 1), ovarian (OE; group 2), deeply infiltrating (DIE; group 3), endometriosis in the cesarean section scar (CC; group 4), and the group of healthy volunteers (HV; group 5). Data are presented as median and interquartile range (Q1–Q3). Table S3: Comparison of the percentage of immune cell subpopulations expressing individual Toll-like receptors (TLR2, TLR3, TLR4, TLR7, TLR8, TLR9) among CD4+ T cells, CD8+ T cells, and CD19+ B cells in patients with different forms of endometriosis: peritoneal (PE; group 1), ovarian (OE; group 2), deeply infiltrating (DIE; group 3), endometriosis in the cesarean section scar (CC; group 4), and healthy volunteers (HV; group 5). Data are presented as median and interquartile range (Q1–Q3). Table S4: Comparison of the concentrations of soluble Toll-like receptors (sTLR2, sTLR3, sTLR4, sTLR7, sTLR8, sTLR9) in serum and urine samples from patients with different forms of endometriosis: peritoneal (PE; group 1), ovarian (OE; group 2), deeply infiltrating (DIE; group 3), endometriosis in the cesarean section scar (CC; group 4), and healthy volunteers (HV; group 5). Data are presented as median and interquartile range (Q1–Q3). Table S5: ROC curve values of the studied parameters of TLR occurrence on individual immune cell subpopulations between individual endometriosis subtypes and healthy volunteers. Table S6: ROC curve values of the studied parameters of sTLR concentration in serum and urine between individual endometriosis subtypes and healthy volunteers.

## Abbreviations

The following abbreviations are used in this manuscript:

BAS	basophils
CC	cesarean section scar endometriosis
CD	cluster of differentiation
CD3	T lymphocytes
CD4	T helper lymphocytes
CD8	cytotoxic T lymphocytes
CD19	B lymphocytes
CD45	total leukocytes
CRP	C-reactive protein
DAMPs	danger-associated molecular patterns
DIE	deeply infiltrating endometriosis
EM	endometriosis
EOS	eosinophils
HGB	hemoglobin
HV	healthy volunteers
IgA	immunoglobulin A
IgG	immunoglobulin G
IgM	immunoglobulin M
IL-6	interleukin-6
IL-8	interleukin-8
LPS	lipopolysaccharides
LYM	lymphocytes
MON	monocytes
MRI	magnetic resonance imaging
MyD88	myeloid differentiation primary response 88
NEU	neutrophils
NF-κB	nuclear factor kappa-light-chain-enhancer of activated B cells
NK	natural killer cells
OE	ovarian endometriosis
PAMPs	pathogen-associated molecular patterns
PE	peritoneal endometriosis
PLT	platelets
PRRs	pattern recognition receptors
ROC	receiver operating characteristic
RBC	red blood cells
sTLR	soluble Toll-like receptor
STAT	signal transducer and activator of transcription
TLR	Toll-like receptor
TLRs	Toll-like receptors
TNF-α	tumor necrosis factor alpha
TRIF	TIR-domain-containing adapter-inducing interferon-β
WBC	white blood cells
