# Supplementary material for: Expression of Toll-like Receptors on Lymphocyte Subpopulations and Their Soluble Forms in Serum and Urine of Women with Endometriosis
Source: Cells. 2025 Aug 18;14(16):1273. doi: 10.3390/cells14161273 (PMC12384095; doi:10.3390/cells14161273)
Supplement: Supplementary file 1 [file cells-14-01273-s001.zip › cells-3759481-supplementary.pdf]

Supplementary Materials Table S1- List of conjugated monoclonal antibodies used for flow cytometric analysis, including fluorochrome conjugates, clones, manufacturers, and catalog numbers.

Antigen	Fluorochrome	Clone	Catalog Number	Manufacturer
CD3	BV510	HIT3a	564713	BD Biosciences
CD4	BV650	SK3	563876	BD Biosciences
CD8	BV605	HIT8a	569169	BD Biosciences
CD16	BV650	3G8	563692	BD Biosciences
CD19	PerCP	SJ25C1	332780	BD Biosciences
CD45	FITC	2D1	345808	BD Biosciences
CD56	BV650	NCAM16.2	564057	BD Biosciences
TLR2	PE	11G7	565349	Biolegend
TLR3	PE	TLR-104	315010	Biolegend
TLR4	APC	HTA125	312816	Biolegend
TLR7	PE	S18024F	376904	Biolegend
TLR8	APC	S16018A	395506	Biolegend
TLR9	APC	S16013D	394808	Biolegend

Supplementary Materials Table S2- Comparison of selected hematological (CRP, RBC, WBC, LYM, MON, BAS, EOS, NEU, HGB, PLT), immunological (IgG, IgM, IgA) parameters, and immune cell subpopulations (CD45+, CD3+, CD4+, CD8+, CD19+) between patients with different forms of endometriosis: peritoneal (PE; group 1), ovarian (OE; group 2), deeply infiltrating (DIE; group 3), endometriosis in the cesarean section scar (CC; group 4), and the group of healthy volunteers (HV; group 5). Data are presented as median and interquartile range (Q1–Q3).

Parameters	PE Median (Q1-Q3)	OE Median (Q1-Q3)	DIE Median (Q1-Q3)	CC Median (Q1-Q3)	HV Median (Q1-Q3)	PE vs. OE	PE vs. DIE	PE vs. CC	PE vs. HV	OE vs. DIE	OE vs. CC	OE vs. HV	DIE vs. CC	DIE vs. HV	CC vs. HV
**CRP [mg/l]**	4,34 (2,90-5,61)	5,65 (3,36-6,49)	7,10 (6,44-7,40)	5,75 (4,35-6,10)	2,76 (1,54-3,32)	>0,9999	0.1075	>0,9999	0.1621	>0,9999	>0,9999	0.0039	>0,9999	<0,0001	0.0012
**RBC [M/ul]**	4,63 (4,40-4,88)	4,32 (3,55-4,54)	4,37 (3,78-4,56)	4,51 (4,43-4,66)	4,53 (4,18-4,64)	0.241	0.6002	>0,9999	>0,9999	>0,9999	>0,9999	>0,9999	>0,9999	>0,9999	>0,9999
**WBC [K/ul]**	6,25 (5,41-7,96)	5,02 (4,81-7,65)	5,84 (4,14-6,92)	6,88 (5,78-7,75)	6,01 (5,71-7,15)	>0,9999	>0,9999	>0,9999	>0,9999	>0,9999	0.4845	>0,9999	>0,9999	>0,9999	>0,9999
**LYM [K/ul]**	1,70 (1,28-2,43)	1,63 (1,52-1,70)	1,91 (1,85-2,38)	1,92 (1,42-2,35)	1,68 (1,39-1,83)	>0,9999	0.2903	>0,9999	>0,9999	0.1101	>0,9999	>0,9999	0.872	0.056	>0,9999

**MON [K/ul]**	0,56 (0,52-0,65)	0,54 (0,41-0,74)	0,41 (0,38-0,44)	0,56 (0,42-0,65)	0,57 (0,49-0,63)	>0,9999	0.0322	>0,9999	>0,9999	0.3749	>0,9999	>0,9999	0.4568	0.1454	>0,9999
**BAS [K/ul]**	0,04 (0,01-0,07)	0,03 (0,03-0,06)	0,04 (0,01-0,05)	0,04 (0,02-0,05)	0,04 (0,03-0,05)	>0,9999	>0,9999	>0,9999	>0,9999	>0,9999	>0,9999	>0,9999	>0,9999	>0,9999	>0,9999
**EOS [K/ul]**	0,02 (0,01-0,06)	0,01 (0,01-0,02)	0,01 (0,01-0,03)	0,02 (0,01-0,06)	0,16 (0,11-0,23)	>0,9999	>0,9999	>0,9999	<0,0001	>0,9999	>0,9999	0.0435	>0,9999	<0,0001	0.0002
**NEU [K/ul]**	4,35(3,87-5,47)	3,77 (2,76-5,27)	5,17 (4,17-5,66)	4,47 (3,50-5,06)	3,26 (2,38-4,16)	>0,9999	>0,9999	>0,9999	0.0197	0.4367	>0,9999	>0,9999	>0,9999	0.0026	0.0584
**HGB [g/dl]**	13,40 (12,35-14,00)	12,75 (9,68-13,20)	13,68 (10,30-14,30)	13,40 (12,90-13,60)	12,90 (12,50-13,80)	0.5893	>0,9999	>0,9999	>0,9999	>0,9999	0.7941	>0,9999	>0,9999	>0,9999	>0,9999
**PLT [K/ul]**	230,00 (199-270)	238,00 (190-402)	291,00 (200-308)	311,00 (271-356)	301,00 (249-302)	>0,9999	>0,9999	0.0234	0.5231	>0,9999	0.4798	>0,9999	>0,9999	>0,9999	>0,9999
**IgG [g/l]**	7,29 (6,48-8,28)	8,05 (7,20-9,29)	7,84 (7,52-8,54)	8,40 (7,27-9,63)	11,81 (8,53-13,55)	>0,9999	>0,9999	>0,9999	<0,0001	>0,9999	>0,9999	0.0525	>0,9999	0.0304	0.0494
**IgM [g/l]**	5,31 (4,72-5,66)	4,26 (3,99-4,51)	3,60 (3,28-3,72)	3,88 (3,49-4,92)	1,59 (1,38-2,33)	0.9483	0.0538	0.217	<0,0001	>0,9999	>0,9999	0.0001	>0,9999	0.0153	0.0001
**IgA [g/l]**	4,96 (4,04-5,89)	6,34 (3,78-6,55)	3,59 (3,47-5,23)	4,70 (4,01-5,93)	1,95 (1,68-2,36)	>0,9999	>0,9999	>0,9999	<0,0001	>0,9999	>0,9999	<0,0001	>0,9999	0.0059	<0,0001
**CD45 [%]**	86,66 (81,78-89,87)	86,38 (81,86-92,64)	90,72 (83,77-94,42)	89,46 (84,62-93,54)	96,28 (92,83-98,05)	>0,9999	>0,9999	>0,9999	<0,0001	>0,9999	>0,9999	0.0015	>0,9999	0.0549	0.0159
**CD3 [%]**	66,02 (61,49-73,58)	67,24 (63,39-73,91)	73,96 (71,42-76,19)	58,86 (55,68-67,55)	72,34 (70,70-76,57)	>0,9999	0.8711	>0,9999	0.0922	>0,9999	0.8158	>0,9999	0.0432	>0,9999	0.0007
**CD4 [%]**	40,99 (32,18-48,95)	53,16 (33,97-55,95)	44,36 (41,27-50,12)	21,44 (20,28-41,51)	47,49 (44,77-49,84)	0.5468	>0,9999	0.8912	0.4384	>0,9999	0.0074	>0,9999	0.2443	>0,9999	0.0015
**CD8 [%]**	24,81 (17,41-31,81)	18,45 (7,33-31,98)	19,78 (17,48-29,56)	20,71 (19,13-22,42)	27,23 (25,88-28,52)	>0,9999	>0,9999	0.8131	>0,9999	>0,9999	>0,9999	0.1294	>0,9999	0.7095	0.0172
**CD19 [%]**	14,01 (10,40-15,13)	13,90 (11,52-14,60)	9,81 (6,54-10,79)	10,68 (10,19-11,42)	12,70 (11,82-13,69)	>0,9999	0.0425	0.0263	>0,9999	0.0878	0.0681	>0,9999	>0,9999	0.0223	0.0108

Supplementary Materials Table S3- Comparison of the percentage of immune cell subpopulations expressing individual Toll-like receptors (TLR2, TLR3, TLR4, TLR7, TLR8, TLR9) among CD4+ T cells, CD8+ T cells, and CD19+ B cells in patients with different forms of endometriosis: peritoneal (PE; group 1), ovarian (OE; group 2), deeply infiltrating (DIE; group 3), endometriosis in the cesarean section scar (CC; group 4), and healthy volunteers (HV; group 5). Data are presented as median and interquartile range (Q1–Q3).

**Parameters**	**PE Median (Q1-Q3)**	**OE Median (Q1-Q3)**	**DIE Median (Q1-Q3)**	**CC Median (Q1-Q3)**	**HV Median (Q1-Q3)**	PE vs. OE	PE vs. DIE	PE vs. CC	PE vs. HV	OE vs. DIE	OE vs. CC	OE vs. HV	DIE vs. CC	DIE vs. HV	CC vs. HV
**CD4+TLR2+**	9,63 (4,38-12,73)	5,22 (2,76-12,26)	3,67 (2,82-4,64)	4,15 (3,48-4,84)	0,81(0,5-0,94)	>0,9999	0.6317	>0,9999	<0,0001	>0,9999	>0,9999	<0,0001	>0,9999	0.0029	<0,0001
**CD4+TLR3+**	10,37 (6,60-13,17)	6,29 (4,84-15,74)	6,50 (4,61-7,01)	16,34 (10,85-19,02)	0,75 (0,20-0,88)	>0,9999	>0,9999	>0,9999	<0,0001	>0,9999	0.3154	0.0021	0.1288	0.0085	<0,0001
**CD4+TLR4+**	6,75 (4,10-11,57)	4,59 (4,36-5,48)	9,79 (6,45-11,28)	3,44 (3,20-3,71)	1,01 (048-1,13)	>0,9999	>0,9999	0.0682	<0,0001	>0,9999	0.6679	<0,0001	0.0323	<0,0001	0.0272
**CD4+TLR7+**	4,46 (2,93-5,27)	5,16 (2,13-5,81)	3,57 (3,38-3,89)	4,69 (2,91-5,24)	0,93 (0,25-1,09)	>0,9999	>0,9999	>0,9999	<0,0001	>0,9999	>0,9999	<0,0001	>0,9999	0.0008	<0,0001
**CD4+TLR8+**	4,24 (3,17-7,09)	3,10 (2,93-6,29)	4,42 (4,03-8,90)	4,67 (4,10-5,74)	0,80 (0,21-0,94)	>0,9999	>0,9999	>0,9999	<0,0001	>0,9999	>0,9999	0.0013	>0,9999	<0,0001	<0,0001
**CD4+TLR9+**	11,20 (6,90-20,23)	7,52 (2,28-19,89)	6,99 (5,09-14,12)	18,19 (16,52-24,90)	0,91 (0,24-1,06)	>0,9999	>0,9999	>0,9999	<0,0001	>0,9999	0.4743	0.002	0.2975	0.0045	<0,0001
**CD8+TLR2+**	14,40 (8,87-19,06)	15,26 (3,87-19,32)	7,27 (6,94-9,04)	17,75 (13,48-22,41)	1,23 (0,75-1,39)	>0,9999	>0,9999	>0,9999	<0,0001	>0,9999	>0,9999	0.0003	0.1438	0.0237	<0,0001
**CD8+TLR3+**	8,32 (5,92-10,69)	5,07 (4,84-7,61)	8,49 (6,89-8,78)	18,52 (15,35-19,81)	0,61 (0,18-0,74)	>0,9999	>0,9999	0.1046	<0,0001	>0,9999	0.0042	0.0707	0.5228	<0,0001	<0,0001
**CD8+TLR4+**	10,84 (8,90-14,56)	10,63-6,24-14,49)	9,08 (8,61-9,56)	6,98 (4,75-11,40)	1,43 (0,76-1,67)	>0,9999	>0,9999	0.9937	<0,0001	>0,9999	>0,9999	<0,0001	>0,9999	0.0002	0.0003
**CD8+TLR7+**	6,83 (4,59-10,69)	9,43 (3,08-12,40)	8,03 (7,61-13,42)	6,82 (4,92-8,35)	1,09 (0,31-1,37)	>0,9999	>0,9999	>0,9999	<0,0001	>0,9999	>0,9999	<0,0001	>0,9999	<0,0001	<0,0001
**CD8+TLR8+**	4,55 (3,24-5,18)	2,98 (2,21-5,41)	5,85 (3,61-7,76)	7,04 (5,29-9,37)	1,00 (0,29-1,25)	>0,9999	>0,9999	0.2794	<0,0001	0.7887	0.095	0.0177	>0,9999	<0,0001	<0,0001
**CD8+TLR9+**	11,90 (7,48-14,62)	11,88 (6,58-12,49)	12,63 (6,89-13,53)	15,54 (12,05-16,11)	1,32 (0,39-1,60)	>0,9999	>0,9999	0.9946	<0,0001	>0,9999	0.448	0.0025	>0,9999	0.0003	<0,0001
**CD19+TLR2+**	12,71 (4,77-14,41)	8,30 (3,27-13,76)	11,58 (9,22-14,04)	19,72 (10,72-22,52)	0,71 (0,42-0,83)	>0,9999	>0,9999	>0,9999	<0,0001	>0,9999	0.294	0.0068	>0,9999	<0,0001	<0,0001

**CD19+TLR3+**	9,67 (5,53-13,14)	6,58 (6,00-13,45)	6,68 (5,22-14,48)	16,03 (15,09-17,51)	0,64 (0,16-0,78)	>0,9999	>0,9999	0.1191	<0,0001	>0,9999	0.1936	0.0016	0.3174	0.0007	<0,0001
**CD19+TLR4+**	7,76 (5,75-14,88)	8,71 (6,36-9,24)	9,05 (7,48-13,09)	9,07 (5,35-14,10)	0,71 (0,35-0,78)	>0,9999	>0,9999	>0,9999	<0,0001	>0,9999	>0,9999	<0,0001	>0,9999	<0,0001	<0,0001
**CD19+TLR7+**	7,58 (4,91-9,01)	3,50 (3,35-9,96)	6,39 (6,09-11,61)	5,67 (4,64-7,60)	0,56 (0,14-0,69)	>0,9999	>0,9999	>0,9999	<0,0001	0.627	>0,9999	0.0074	>0,9999	<0,0001	<0,0001
**CD19+TLR8+**	5,96 (5,28-8,62)	6,34 (1,32-8,77)	11,43 (10,77-11,95)	5,46 (4,95-7,04)	0,51 (0,13-0,62)	>0,9999	0.3865	>0,9999	<0,0001	0.2823	>0,9999	0.0009	0.1907	<0,0001	0.0001
**CD19+TLR9+**	9,45 (7,03-10,20)	7,67 (6,90-9,00)	8,87 (6,99-9,99)	13,42 (12,33-16,30)	0,45 (0,11-0,55)	>0,9999	>0,9999	0.4499	<0,0001	>0,9999	0.1109	0.0091	>0,9999	0.0002	<0,0001

Supplementary Materials Table S4- Comparison of the concentrations of soluble Toll-like receptors (sTLR2, sTLR3, sTLR4, sTLR7, sTLR8, sTLR9) in serum and urine samples from patients with different forms of endometriosis: peritoneal (PE; group 1), ovarian (OE; group 2), deeply infiltrating (DIE; group 3), endometriosis in the cesarean section scar (CC; group 4), and healthy volunteers (HV; group 5). Data are presented as median and interquartile range (Q1–Q3).

**Parameters**	**PE (group 1) Median (Q1-Q3)**	**OE (group 2) Median (Q1-Q3)**	**DIE (group 3) Median (Q1-Q3)**	**CC (group 4) Median (Q1-Q3)**	**HV (group 5) Median (Q1-Q3)**	PE vs. OE	PE vs. DIE	PE vs. CC	PE vs. HV	OE vs. DIE	OE vs. CC	OE vs. HV	DIE vs. CC	DIE vs. HV	CC vs. HV
**Serum sTLR2**	18,84 (16,38-21,02)	20,28 (19,06-22,84)	17,63 (16,94-20,21)	17,25 (16,27-20,27)	2,92 (2,033-3,72)	>0,9999	>0,9999	>0,9999	<0,0001	>0,9999	>0,9999	<0,0001	>0,9999	0.0002	<0,0001
**Serum sTLR3**	12,04 (11,25-13,82)	14,06 (13,21-14,40)	13,50 (12,52-14,50)	13,27 (12,40-14,04)	2,33 (1,78-2,58)	>0,9999	>0,9999	>0,9999	<0,0001	>0,9999	>0,9999	<0,0001	>0,9999	<0,0001	<0,0001
**Serum sTLR4**	19,34 (17,69-21,81)	17,83 (17,18-18,28)	20,32 (15,64-22,28)	20,71 (18,52-21,95)	3,59 (3,04-4,14)	>0,9999	>0,9999	>0,9999	<0,0001	>0,9999	>0,9999	0.0022	>0,9999	<0,0001	<0,0001
**Serum sTLR7**	10,12 (9,23-11,05)	9,43 (8,59-11,42)	10,01 (8,98-10,73)	10,19 (8,84-10,65)	2,83 (2,07-3,28)	>0,9999	>0,9999	>0,9999	<0,0001	>0,9999	>0,9999	0.0002	>0,9999	<0,0001	<0,0001
**Serum sTLR8**	10,05 (9,41-11,59)	11,62 (10,87-12,26)	10,61 (9,94-12,38)	10,34 (8,89-12,05)	1,69 (1,42-1,82)	>0,9999	>0,9999	>0,9999	<0,0001	>0,9999	>0,9999	<0,0001	>0,9999	<0,0001	<0,0001
**Serum sTLR9**	14,01 (11,31-16,16)	16,36 (10,82-18,29)	13,07 (11,05-16,22)	14,19 (11,62-17,12)	3,52 (2,63-4,45)	>0,9999	>0,9999	>0,9999	<0,0001	>0,9999	>0,9999	<0,0001	>0,9999	0.0005	<0,0001
**Urine sTLR2**	3,92 (2,73-4,33)	3,48 (2,59-3,70)	3,83 (3,55-4,72)	4,38 (2,80-5,29)	1,37 (1,03-1,79)	>0,9999	>0,9999	>0,9999	<0,0001	>0,9999	>0,9999	0.0165	>0,9999	<0,0001	<0,0001

**Urine sTLR3**	3,19 (2,43-3,84)	3,14 (2,83-3,38)	4,44 (2,94-4,61)	4,32 (3,58-4,60)	0,89 (0,78-1,14)	>0,9999	>0,9999	0.574	<0,0001	>0,9999	0.9222	0.0015	>0,9999	<0,0001	<0,0001
**Urine sTLR4**	5,50 (4,87-6,08)	6,92 (6,46-7,29)	5,37 (5,23-5,92)	5,29 (4,46-5,92)	1,62 (1,35-2,03)	0.184	>0,9999	>0,9999	<0,0001	0.4646	0.1026	<0,0001	>0,9999	0.0002	0.0002
**Urine sTLR7**	2,18 (1,82-2,53)	1,20 (1,04-1,96)	2,30 (1,85-2,84)	1,82 (1,33-2,51)	1,27 (0,85-1,64)	0.023	>0,9999	>0,9999	0.0007	0.0122	0.3508	>0,9999	>0,9999	0.0006	0.0557
**Urine sTLR8**	2,91 (2,37-3,51)	2,17 (1,04-3,88)	1,99 (1,30-2,30)	2,44 (1,93-3,05)	0,71 (0,57-0,93)	>0,9999	0.4709	>0,9999	<0,0001	>0,9999	>0,9999	0.0011	>0,9999	0.0117	<0,0001
**Urine sTLR9**	4,63 (2,72-5,22)	4,98 (4,34-5,26)	3,87 (3,62-4,41)	3,08 (2,73-3,95)	1,78 (1,22-1,98)	>0,9999	>0,9999	>0,9999	<0,0001	>0,9999	0.1177	<0,0001	>0,9999	0.0002	0.0047

Supplementary Materials Table S5- ROC curve values of the studied parameters of TLR occurrence on individual immune cell subpopulations between individual endometriosis subtypes and healthy volunteers

	CD4+TLR2+ [%]									
	PE vs. HV	PE vs. OE	PE vs. DIE	PE vs. CC	OE vs. DIE	OE vs. CC	OE vs. HV	DIE vs CC	DIE vs. HV	CC vs. HV
Area	1	0,6458	0,8	0,779	0,7333	0,619	1	0,6143	1	1
Std. Error	0	0,1773	0,1039	0,0927	0,1947	0,2581	0	0,1776	0	0
95% confidence interval	1,000 to 1,000	0,2982 to 0,9934	0,5963 to 1,000	0,5973 to 0,9607	0,3516 to 1,000	0,1132 to 1,000	1,000 to 1,000	0,2662 to 0,9624	1,000 to 1,000	1,000 to 1,000
P value	<0,0001	0,4338	0,0475	0,0094	0,2967	0,5287	0,0055	0,4589	0,0005	<0,0001
	CD4+TLR3+ [%]									
	PE vs. HV	PE vs. OE	PE vs. DIE	PE vs. CC	OE vs. DIE	OE vs. CC	OE vs. HV	DIE vs CC	DIE vs. HV	CC vs. HV
Area	1	0,625	0,7375	0,7768	0,6	0,8333	1	0,9143	1	1
Std. Error	0	0,2266	0,1451	0,08505	0,2271	0,1477	0	0,08448	0	0
95% confidence interval	1,000 to 1,000	0,1808 to 1,000	0,4531 to 1,000	0,6101 to 0,9435	0,1550 to 1,000	0,5438 to 1,000	1,000 to 1,000	0,7487 to 1,000	1,000 to 1,000	1,000 to 1,000
P value	<0,0001	0,5023	0,1167	0,01	0,6547	0,0778	0,0055	0,0073	0,0005	<0,0001
	CD4+TLR4+ [%]									
	PE vs. HV	PE vs. OE	PE vs. DIE	PE vs. CC	OE vs. DIE	OE vs. CC	OE vs. HV	DIE vs CC	DIE vs. HV	CC vs. HV
Area	1	0,625	0,5625	0,8594	0,8	1	1	0,9857	1	1
Std. Error	0	0,1194	0,1335	0,07956	0,1789	0	0	0,02246	0	0
95% confidence interval	1,000 to 1,000	0,3911 to 0,8589	0,3008 to 0,8242	0,7034 to 1,000	0,4494 to 1,000	1,000 to 1,000	1,000 to 1,000	0,9417 to 1,000	1,000 to 1,000	1,000 to 1,000
P value	<0,0001	0,5023	0,6797	0,0008	0,1797	0,0082	0,0055	0,0016	0,0005	<0,0001

	CD4+TLR7+ [%]									
	PE vs. HV	PE vs. OE	PE vs. DIE	PE vs. CC	OE vs. DIE	OE vs. CC	OE vs. HV	DIE vs CC	DIE vs. HV	CC vs. HV
Area	1	0,5625	0,6625	0,5201	0,6667	0,619	1	0,5857	1	1
Std. Error	0	0,2032	0,1268	0,1082	0,2271	0,2012	0	0,1342	0	0
95% confidence interval	1,000 to 1,000	0,1643 to 0,9607	0,4140 to 0,9110	0,3079 to 0,7322	0,2216 to 1,000	0,2248 to 1,000	1,000 to 1,000	0,3228 to 0,8486	1,000 to 1,000	1,000 to 1,000
P value	<0,0001	0,7373	0,2831	0,8516	0,4561	0,5287	0,0055	0,5786	0,0005	<0,0001
	CD4+TLR8+ [%]									
	PE vs. HV	PE vs. OE	PE vs. DIE	PE vs. CC	OE vs. DIE	OE vs. CC	OE vs. HV	DIE vs CC	DIE vs. HV	CC vs. HV
Area	1	0,6042	0,5875	0,5379	0,6667	0,6429	1	0,5571	1	1
Std. Error	0	0,1632	0,1608	0,11	0,2051	0,2405	0	0,1837	0	0
95% confidence interval	1,000 to 1,000	0,2843 to 0,9240	0,2723 to 0,9027	0,3224 to 0,7535	0,2646 to 1,000	0,1714 to 1,000	1,000 to 1,000	0,1971 to 0,9172	1,000 to 1,000	1,000 to 1,000
P value	<0,0001	0,5762	0,5633	0,7238	0,4561	0,4497	0,0055	0,7111	0,0005	<0,0001
	CD4+TLR9+ [%]									
	PE vs. HV	PE vs. OE	PE vs. DIE	PE vs. CC	OE vs. DIE	OE vs. CC	OE vs. HV	DIE vs CC	DIE vs. HV	CC vs. HV
Area	1	0,625	0,675	0,721	0,5333	0,7619	1	0,9286	1	1
Std. Error	0	0,1983	0,1356	0,09639	0,2553	0,1782	0	0,05947	0	0
95% confidence interval	1,000 to 1,000	0,2363 to 1,000	0,4092 to 0,9408	0,5321 to 0,9099	0,03293 to 1,000	0,4125 to 1,000	1,000 to 1,000	0,8120 to 1,000	1,000 to 1,000	1,000 to 1,000
P value	<0,0001	0,5023	0,2477	0,0396	0,8815	0,1658	0,0055	0,0055	0,0005	<0,0001
	CD8+TLR2+ [%]									
	PE vs. HV	PE vs. OE	PE vs. DIE	PE vs. CC	OE vs. DIE	OE vs. CC	OE vs. HV	DIE vs CC	DIE vs. HV	CC vs. HV
Area	1	0,5417	0,8	0,6674	0,7333	0,6905	1	0,9571	1	1
Std. Error	0	0,1912	0,09582	0,09973	0,2309	0,1728	0	0,04832	0	0
95% confidence interval	1,000 to 1,000	0,1669 to 0,9165	0,6122 to 0,9878	0,4719 to 0,8629	0,2807 to 1,000	0,3517 to 1,000	1,000 to 1,000	0,8624 to 1,000	1,000 to 1,000	1,000 to 1,000
P value	<0,0001	0,8231	0,0475	0,119	0,2967	0,3135	0,0055	0,0031	0,0005	<0,0001
	CD8+TLR3+ [%]									
	PE vs. HV	PE vs. OE	PE vs. DIE	PE vs. CC	OE vs. DIE	OE vs. CC	OE vs. HV	DIE vs CC	DIE vs. HV	CC vs. HV
Area	1	0,75	0,55	0,9576	0,8667	1	1	0,8714	1	1
Std. Error	0	0,1151	0,1459	0,03373	0,144	0	0	0,12	0	0
95% confidence interval	1,000 to 1,000	0,5245 to 0,9755	0,2641 to 0,8359	0,8915 to 1,000	0,5844 to 1,000	1,000 to 1,000	1,000 to 1,000	0,6362 to 1,000	1,000 to 1,000	1,000 to 1,000
P value	<0,0001	0,1797	0,7412	<0,0001	0,1011	0,0082	0,0055	0,0161	0,0005	<0,0001

	CD8+TLR4+ [5]									
	PE vs. HV	PE vs. OE	PE vs. DIE	PE vs. CC	OE vs. DIE	OE vs. CC	OE vs. HV	DIE vs CC	DIE vs. HV	CC vs. HV
Area	1	0,5833	0,7125	0,7344	0,7333	0,7381	1	0,6286	1	1
Std. Error	0	0,1787	0,1122	0,09393	0,2309	0,1466	0	0,125	0	0
95% confidence interval	1,000 to 1,000	0,2331 to 0,9336	0,4926 to 0,9324	0,5503 to 0,9185	0,2807 to 1,000	0,4507 to 1,000	1,000 to 1,000	0,3836 to 0,8736	1,000 to 1,000	1,000 to 1,000
P value	<0,0001	0,6547	0,1604	0,0291	0,2967	0,2077	0,0055	0,4047	0,0005	<0,0001
	CD8+TLR7+ [%]									
	PE vs. HV	PE vs. OE	PE vs. DIE	PE vs. CC	OE vs. DIE	OE vs. CC	OE vs. HV	DIE vs CC	DIE vs. HV	CC vs. HV
Area	1	0,5417	0,625	0,5513	0,5333	0,6667	1	0,6857	1	1
Std. Error	0	0,212	0,1578	0,1099	0,2177	0,2722	0	0,1726	0	0
95% confidence interval	1,000 to 1,000	0,1262 to 0,9572	0,3157 to 0,9343	0,3360 to 0,7667	0,1066 to 0,9601	0,1332 to 1,000	1,000 to 1,000	0,3473 to 1,000	1,000 to 1,000	1,000 to 1,000
P value	<0,0001	0,8231	0,409	0,6326	0,8815	0,3778	0,0055	0,2288	0,0005	<0,0001
	CD8+TLR8+ [%]									
	PE vs. HV	PE vs. OE	PE vs. DIE	PE vs. CC	OE vs. DIE	OE vs. CC	OE vs. HV	DIE vs CC	DIE vs. HV	CC vs. HV
Area	1	0,6042	0,7125	0,8817	0,8667	0,881	1	0,6286	1	1
Std. Error	0	0,2002	0,1611	0,06051	0,144	0,1117	0	0,1755	0	0
95% confidence interval	1,000 to 1,000	0,2118 to 0,9965	0,3968 to 1,000	0,7631 to 1,000	0,5844 to 1,000	0,6620 to 1,000	1,000 to 1,000	0,2846 to 0,9725	1,000 to 1,000	1,000 to 1,000
P value	<0,0001	0,5762	0,1604	0,0004	0,1011	0,0438	0,0055	0,4047	0,0005	<0,0001
	CD8+TLR9+ [%]									
	PE vs. HV	PE vs. OE	PE vs. DIE	PE vs. CC	OE vs. DIE	OE vs. CC	OE vs. HV	DIE vs CC	DIE vs. HV	CC vs. HV
Area	1	0,5625	0,5063	0,7522	0,6667	0,8333	1	0,8143	1	1
Std. Error	0	0,1468	0,1457	0,09146	0,2022	0,107	0	0,1031	0	0
95% confidence interval	1,000 to 1,000	0,2747 to 0,8503	0,2207 to 0,7918	0,5730 to 0,9315	0,2703 to 1,000	0,6235 to 1,000	1,000 to 1,000	0,6122 to 1,000	1,000 to 1,000	1,000 to 1,000
P value	<0,0001	0,7373	0,9671	0,0188	0,4561	0,0778	0,0055	0,0417	0,0005	<0,0001
	CD19+TLR2+ [%]									
	PE vs. HV	PE vs. OE	PE vs. DIE	PE vs. CC	OE vs. DIE	OE vs. CC	OE vs. HV	DIE vs CC	DIE vs. HV	CC vs. HV
Area	1	0,625	0,525	0,7522	0,6667	0,8333	1	0,7286	1	1
Std. Error	0	0,1609	0,1376	0,09102	0,2271	0,1275	0	0,1198	0	0
95% confidence interval	1,000 to 1,000	0,3096 to 0,9404	0,2553 to 0,7947	0,5738 to 0,9306	0,2216 to 1,000	0,5834 to 1,000	1,000 to 1,000	0,4937 to 0,9634	1,000 to 1,000	1,000 to 1,000
P value	<0,0001	0,5023	0,8688	0,0188	0,4561	0,0778	0,0055	0,1385	0,0005	<0,0001

	CD19+TLR3+ [%]									
	PE vs. HV	PE vs. OE	PE vs. DIE	PE vs. CC	OE vs. DIE	OE vs. CC	OE vs. HV	DIE vs CC	DIE vs. HV	CC vs. HV
Area	1	0,5	0,5375	0,9263	0,6	0,9524	1	0,8	1	1
Std. Error	0	0,1712	0,1752	0,0481	0,208	0,05596	0	0,1535	0	0
95% confidence interval	1,000 to 1,000	0,1645 to 0,8355	0,1941 to 0,8809	0,8321 to 1,000	0,1923 to 1,000	0,8427 to 1,000	1,000 to 1,000	0,4992 to 1,000	1,000 to 1,000	1,000 to 1,000
P value	<0,0001	>0,9999	0,8044	<0,0001	0,6547	0,0167	0,0055	0,0519	0,0005	<0,0001
	CD19+TLR4+ [%]									
	PE vs. HV	PE vs. OE	PE vs. DIE	PE vs. CC	OE vs. DIE	OE vs. CC	OE vs. HV	DIE vs CC	DIE vs. HV	CC vs. HV
Area	1	0,5	0,5875	0,5067	0,6	0,5238	1	0,5286	1	1
Std. Error	0	0,1412	0,1509	0,1084	0,208	0,1342	0	0,1663	0	0
95% confidence interval	1,000 to 1,000	0,2232 to 0,7768	0,2918 to 0,8832	0,2942 to 0,7191	0,1923 to 1,000	0,2608 to 0,7868	1,000 to 1,000	0,2026 to 0,8546	1,000 to 1,000	1,000 to 1,000
P value	<0,0001	>0,9999	0,5633	0,9503	0,6547	0,8997	0,0055	0,8531	0,0005	<0,0001
	CD19+TLR7+ [%]									
	PE vs. HV	PE vs. OE	PE vs. DIE	PE vs. CC	OE vs. DIE	OE vs. CC	OE vs. HV	DIE vs CC	DIE vs. HV	CC vs. HV
Area	1	0,6875	0,6	0,6183	0,8	0,6905	1	0,7429	1	1
Std. Error	0	0,2237	0,1599	0,1053	0,1886	0,2545	0	0,1303	0	0
95% confidence interval	1,000 to 1,000	0,2491 to 1,000	0,2866 to 0,9134	0,4120 to 0,8246	0,4304 to 1,000	0,1918 to 1,000	1,000 to 1,000	0,4875 to 0,9982	1,000 to 1,000	1,000 to 1,000
P value	<0,0001	0,3143	0,5089	0,2706	0,1797	0,3135	0,0055	0,1155	0,0005	<0,0001
	CD19+TLR8+ [%]									
	PE vs. HV	PE vs. OE	PE vs. DIE	PE vs. CC	OE vs. DIE	OE vs. CC	OE vs. HV	DIE vs CC	DIE vs. HV	CC vs. HV
Area	1	0,5417	0,8875	0,5714	0,8667	0,5238	1	0,9143	1	1
Std. Error	0	0,2071	0,1005	0,1087	0,1398	0,2338	0	0,08448	0	0
95% confidence interval	1,000 to 1,000	0,1357 to 0,9477	0,6906 to 1,000	0,3584 to 0,7845	0,5926 to 1,000	0,06556 to 0,9821	1,000 to 1,000	0,7487 to 1,000	1,000 to 1,000	1,000 to 1,000
P value	<0,0001	0,8231	0,0105	0,506	0,1011	0,8997	0,0055	0,0073	0,0005	<0,0001
	CD19+TLR9+ [%]									
	PE vs. HV	PE vs. OE	PE vs. DIE	PE vs. CC	OE vs. DIE	OE vs. CC	OE vs. HV	DIE vs CC	DIE vs. HV	CC vs. HV
Area	1	0,6354	0,5	0,8594	0,6	0,9286	1	0,7429	1	1
Std. Error	0	0,1734	0,1532	0,07839	0,2297	0,06883	0	0,1754	0	0
95% confidence interval	1,000 to 1,000	0,2955 to 0,9753	0,1998 to 0,8002	0,7057 to 1,000	0,1499 to 1,000	0,7937 to 1,000	1,000 to 1,000	0,3990 to 1,000	1,000 to 1,000	1,000 to 1,000
P value	<0,0001	0,4674	>0,9999	0,0008	0,6547	0,0233	0,0055	0,1155	0,0005	<0,0001

Supplementary Materials Table S6- ROC curve values of the studied parameters of sTLR concentration in serum and urine between individual endometriosis subtypes and healthy volunteers

	Serum concentration of sTLR2									
	PE vs. HV	PE vs. OE	PE vs. DIE	PE vs. CC	OE vs. DIE	OE vs. CC	OE vs. HV	DIE vs CC	DIE vs. HV	CC vs. HV
Area	1	0,7708	0,525	0,567	0,7333	0,8333	1	0,5857	1	1
Std. Error	0	0,1143	0,135	0,1075	0,1917	0,09906	0	0,145	0	0
95% confidence interval	1,000 to 1,000	0,5468 to 0,9949	0,2605 to 0,7895	0,3563 to 0,7776	0,3576 to 1,000	0,6392 to 1,000	1,000 to 1,000	0,3016 to 0,8699	1,000 to 1,000	1,000 to 1,000
P value	<0,0001	0,1461	0,8688	0,5329	0,2967	0,0778	0,0055	0,5786	0,0005	<0,0001
	Serum concentration of sTLR3									
	PE vs. HV	PE vs. OE	PE vs. DIE	PE vs. CC	OE vs. DIE	OE vs. CC	OE vs. HV	DIE vs CC	DIE vs. HV	CC vs. HV
Area	1	0,8125	0,65	0,6429	0,6	0,7619	1	0,5714	1	1
Std. Error	0	0,1023	0,1599	0,1032	0,2191	0,1259	0	0,1752	0	0
95% confidence interval	1,000 to 1,000	0,6120 to 1,000	0,3366 to 0,9634	0,4405 to 0,8452	0,1706 to 1,000	0,5152 to 1,000	1,000 to 1,000	0,2280 to 0,9148	1,000 to 1,000	1,000 to 1,000
P value	<0,0001	0,0935	0,3218	0,1834	0,6547	0,1658	0,0055	0,6434	0,0005	<0,0001
	Serum concentration of sTLR4									
	PE vs. HV	PE vs. OE	PE vs. DIE	PE vs. CC	OE vs. DIE	OE vs. CC	OE vs. HV	DIE vs CC	DIE vs. HV	CC vs. HV
Area	1	0,6667	0,5125	0,5625	0,5333	0,7024	1	0,5429	1	1
Std. Error	0	0,1521	0,1809	0,108	0,215	0,1287	0	0,173	0	0
95% confidence interval	1,000 to 1,000	0,3686 to 0,9647	0,1579 to 0,8671	0,3509 to 0,7741	0,1120 to 0,9547	0,4501 to 0,9546	1,000 to 1,000	0,2038 to 0,8819	1,000 to 1,000	1,000 to 1,000
P value	<0,0001	0,3711	0,9342	0,5606	0,8815	0,2842	0,0055	0,7812	0,0005	<0,0001
	Serum concentration of sTLR7									
	PE vs. HV	PE vs. OE	PE vs. DIE	PE vs. CC	OE vs. DIE	OE vs. CC	OE vs. HV	DIE vs CC	DIE vs. HV	CC vs. HV
Area	1	0,5208	0,5625	0,558	0,5333	0,5	1	0,5286	1	1
Std. Error	0	0,2201	0,1483	0,1087	0,253	0,2309	0	0,151	0	0
95% confidence interval	1,000 to 1,000	0,08939 to 0,9523	0,2719 to 0,8531	0,3451 to 0,7710	0,03750 to 1,000	0,04750 to 0,9525	1,000 to 1,000	0,2326 to 0,8245	1,000 to 1,000	1,000 to 1,000
P value	<0,0001	0,911	0,6797	0,5889	0,8815	>0,9999	0,0055	0,8531	0,0005	<0,0001
	Serum concentration of sTLR8									
	PE vs. HV	PE vs. OE	PE vs. DIE	PE vs. CC	OE vs. DIE	OE vs. CC	OE vs. HV	DIE vs CC	DIE vs. HV	CC vs. HV
Area	1	0,8125	0,6313	0,5246	0,6667	0,7143	1	0,6143	1	1
Std. Error	0	0,1011	0,1528	0,1117	0,2022	0,1244	0	0,144	0	0

95% confidence interval	1,000 to 1,000	0,6143 to 1,000	0,3317 to 0,9308	0,3055 to 0,7436	0,2703 to 1,000	0,4705 to 0,9580	1,000 to 1,000	0,3320 to 0,8965	1,000 to 1,000	1,000 to 1,000
P value	<0,0001	0,0935	0,3859	0,8192	0,4561	0,2568	0,0055	0,4589	0,0005	<0,0001
	Serum concentration of sTLR9									
	PE vs. HV	PE vs. OE	PE vs. DIE	PE vs. CC	OE vs. DIE	OE vs. CC	OE vs. HV	DIE vs CC	DIE vs. HV	CC vs. HV
Area	1	0,6875	0,525	0,5625	0,7333	0,6429	1	0,6286	1	1
Std. Error	0	0,2565	0,1625	0,1101	0,2309	0,2412	0	0,1396	0	0
95% confidence interval	1,000 to 1,000	0,1848 to 1,000	0,2066 to 0,8434	0,3467 to 0,7783	0,2807 to 1,000	0,1702 to 1,000	1,000 to 1,000	0,3549 to 0,9023	1,000 to 1,000	1,000 to 1,000
P value	<0,0001	0,3143	0,8688	0,5606	0,2967	0,4497	0,0055	0,4047	0,0005	<0,0001
	Urine concentration of sTLR2									
	PE vs. HV	PE vs. OE	PE vs. DIE	PE vs. CC	OE vs. DIE	OE vs. CC	OE vs. HV	DIE vs CC	DIE vs. HV	CC vs. HV
Area	1	0,625	0,625	0,6295	0,8	0,6905	1	0,5143	1	1
Std. Error	0	0,1239	0,1317	0,1081	0,1651	0,1231	0	0,1511	0	0
95% confidence interval	1,000 to 1,000	0,3821 to 0,8679	0,3668 to 0,8832	0,4176 to 0,8414	0,4764 to 1,000	0,4492 to 0,9318	1,000 to 1,000	0,2181 to 0,8105	1,000 to 1,000	1,000 to 1,000
P value	<0,0001	0,5023	0,409	0,228	0,1797	0,3135	0,0055	0,9262	0,0005	<0,0001
	Urine concentration of sTLR3									
	PE vs. HV	PE vs. OE	PE vs. DIE	PE vs. CC	OE vs. DIE	OE vs. CC	OE vs. HV	DIE vs CC	DIE vs. HV	CC vs. HV
Area	1	0,5208	0,725	0,7857	0,7	0,8571	1	0,5714	1	1
Std. Error	0	0,1433	0,1369	0,08576	0,1956	0,09967	0	0,1584	0	0
95% confidence interval	1,000 to 1,000	0,2400 to 0,8017	0,4567 to 0,9933	0,6176 to 0,9538	0,3167 to 1,000	0,6618 to 1,000	1,000 to 1,000	0,2610 to 0,8818	1,000 to 1,000	1,000 to 1,000
P value	<0,0001	0,911	0,1372	0,0078	0,3711	0,0588	0,0055	0,6434	0,0005	<0,0001
	Urine concentration of sTLR4									
	PE vs. HV	PE vs. OE	PE vs. DIE	PE vs. CC	OE vs. DIE	OE vs. CC	OE vs. HV	DIE vs CC	DIE vs. HV	CC vs. HV
Area	1	0,9271	0,5625	0,5558	0,9333	0,9286	1	0,6143	1	1
Std. Error	0	0,06178	0,1312	0,1076	0,09428	0,06541	0	0,1293	0	0
95% confidence interval	1,000 to 1,000	0,8060 to 1,000	0,3054 to 0,8196	0,3449 to 0,7667	0,7485 to 1,000	0,8004 to 1,000	1,000 to 1,000	0,3609 to 0,8676	1,000 to 1,000	1,000 to 1,000
P value	<0,0001	0,0219	0,6797	0,6033	0,0526	0,0233	0,0055	0,4589	0,0005	<0,0001
	Urine concentration of sTLR7									
	PE vs. HV	PE vs. OE	PE vs. DIE	PE vs. CC	OE vs. DIE	OE vs. CC	OE vs. HV	DIE vs CC	DIE vs. HV	CC vs. HV
Area	1	0,8958	0,6063	0,6004	0,8667	0,7262	1	0,6857	1	1
Std. Error	0	0,09859	0,1602	0,1084	0,144	0,1518	0	0,1306	0	0

95% confidence interval	1,000 to 1,000	0,7026 to 1,000	0,2923 to 0,9202	0,3880 to 0,8129	0,5844 to 1,000	0,4287 to 1,000	1,000 to 1,000	0,4298 to 0,9416	1,000 to 1,000	1,000 to 1,000
P value	<0,0001	0,0336	0,4828	0,3496	0,1011	0,2314	0,0055	0,2288	0,0005	<0,0001
	Urine concentration of sTLR8									
	PE vs. HV	PE vs. OE	PE vs. DIE	PE vs. CC	OE vs. DIE	OE vs. CC	OE vs. HV	DIE vs CC	DIE vs. HV	CC vs. HV
Area	1	0,625	0,875	0,6473	0,5333	0,5476	1	0,7429	1	1
Std. Error	0	0,2593	0,07834	0,1033	0,2553	0,2452	0	0,1237	0	0
95% confidence interval	1,000 to 1,000	0,1168 to 1,000	0,7215 to 1,000	0,4449 to 0,8498	0,03293 to 1,000	0,06711 to 1,000	1,000 to 1,000	0,5004 to 0,9853	1,000 to 1,000	1,000 to 1,000
P value	<0,0001	0,5023	0,0132	0,1701	0,8815	0,8011	0,0055	0,1155	0,0005	<0,0001
	Urine concentration of sTLR9									
	PE vs. HV	PE vs. OE	PE vs. DIE	PE vs. CC	OE vs. DIE	OE vs. CC	OE vs. HV	DIE vs CC	DIE vs. HV	CC vs. HV
Area	1	0,6667	0,5375	0,6518	0,9333	0,9286	1	0,7571	1	1
Std. Error	0	0,1229	0,1225	0,105	0,09428	0,06883	0	0,1134	0	0
95% confidence interval	1,000 to 1,000	0,4258 to 0,9075	0,2974 to 0,7776	0,4461 to 0,8575	0,7485 to 1,000	0,7937 to 1,000	1,000 to 1,000	0,5349 to 0,9794	1,000 to 1,000	1,000 to 1,000
P value	<0,0001	0,3711	0,8044	0,1575	0,0526	0,0233	0,0055	0,0956	0,0005	<0,0001

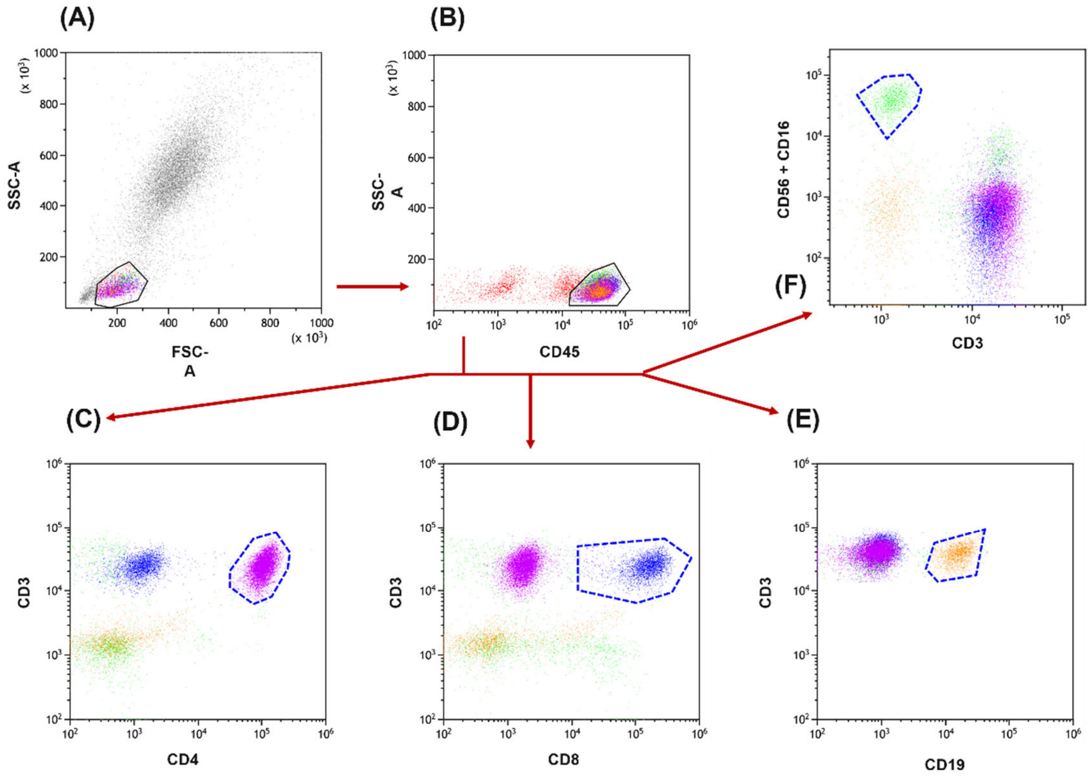

Supplementary Materials Figure S1. Gating strategy for lymphocyte subpopulations. (A) Forward scatter (FSC-A) versus side scatter (SSC-A) plot for initial lymphocyte gating. (B) CD45 versus SSC-A plot to define leukocytes. (C) CD3 vs CD4 plot for CD4+ T cells, (violet) (D) CD3 vs CD8 plot for CD8⁺ T cells, (blue) (E) CD3 vs CD19 plot for B cells (orange), (F) CD3 vs CD56+CD16 plot to define natural killer (NK) cells (green), characterized by CD3⁻/CD56⁺CD16⁺ phenotype.

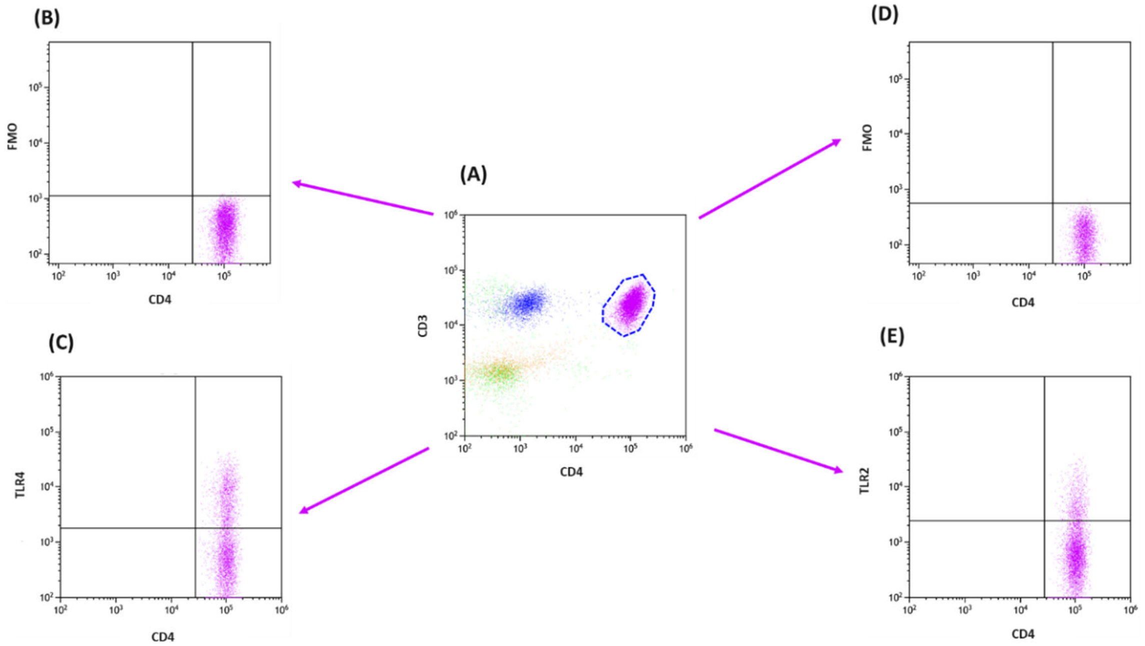

Supplementary Materials Figure S2. The figure shows the analysis of TLR2 and TLR4 expression on CD4⁺ T cells using flow cytometry, including a complete gating strategy and quality control of the assays. Panel (A) illustrates the CD4⁺ T cell population selected based on the simultaneous expression of the CD3 and CD4 surface markers. Fluorescence minus one (FMO) controls (shown in panels (B) and (D) were used to correctly define the fluorescence threshold and define the boundaries of positive expression; FMO controls for the PE dye (for TLR2) and the APC dye (for TLR4) distinguish the specific signal from autofluorescence and background. Panel (C) shows the level of TLR4 expression (APC dye) within the selected CD4⁺ cell population, while panel (E) shows the expression of TLR2 (PE dye) in the same population. The use of two independent FMO controls for different fluorescence channels allowed for a reliable assessment of the presence of TLRs on CD4⁺ T lymphocytes, minimizing the risk of misinterpretation of results resulting from overlapping fluorescence spectra or inhomogeneous background.

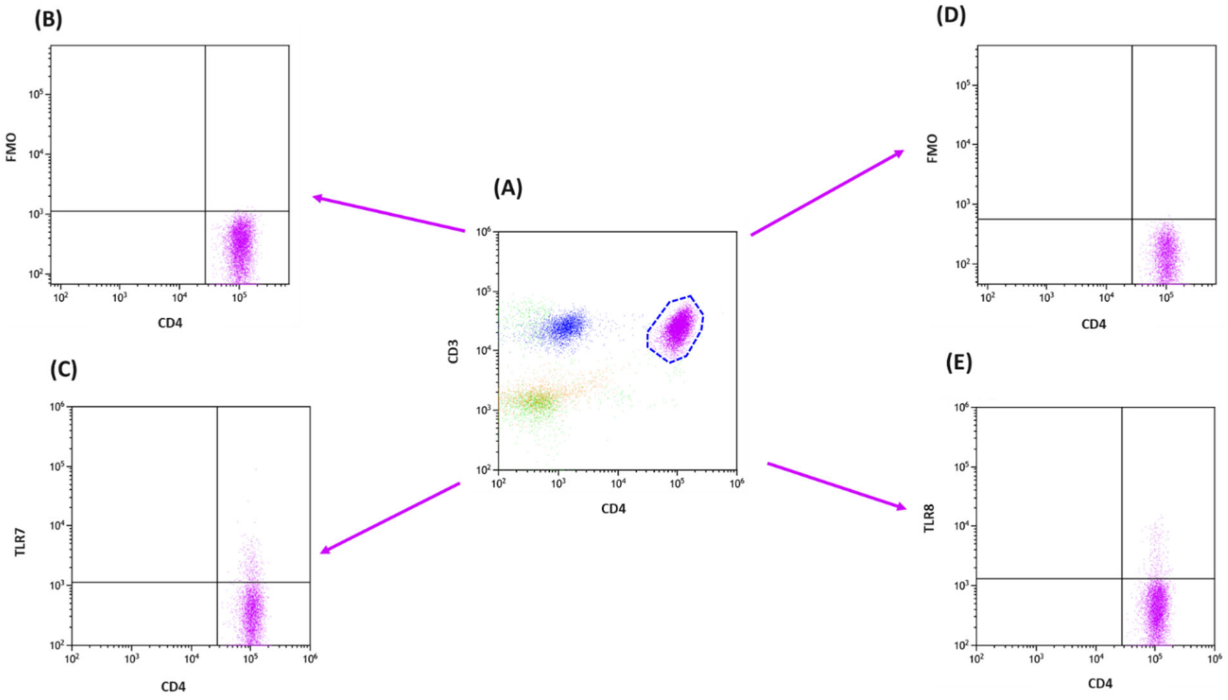

Supplementary Materials Figure S3. The figure shows the analysis of TLR7 and TLR8 expression on CD4$^+$ T lymphocytes using flow cytometry and fluorescence signal quality control. Panel (A) shows the gating of the CD3$^+$CD4$^+$ lymphocyte population, which is the starting point for further analyses. Panels (B) and (D) show FMO (fluorescence minus one) controls, respectively for the PE (TLR7) and APC (TLR8) dyes, which allow for precise determination of the positive fluorescence limit and elimination of errors resulting from autofluorescence and background. Panel (C) shows the level of TLR7 expression, while panel (E) shows TLR8 expression on the surface of CD4$^+$ lymphocytes. The use of two independent FMO controls adapted to a specific fluorescence channel allowed for precise and reliable determination of the percentage of cells expressing the analyzed Toll-like receptors, increasing the accuracy and reproducibility of the obtained results.

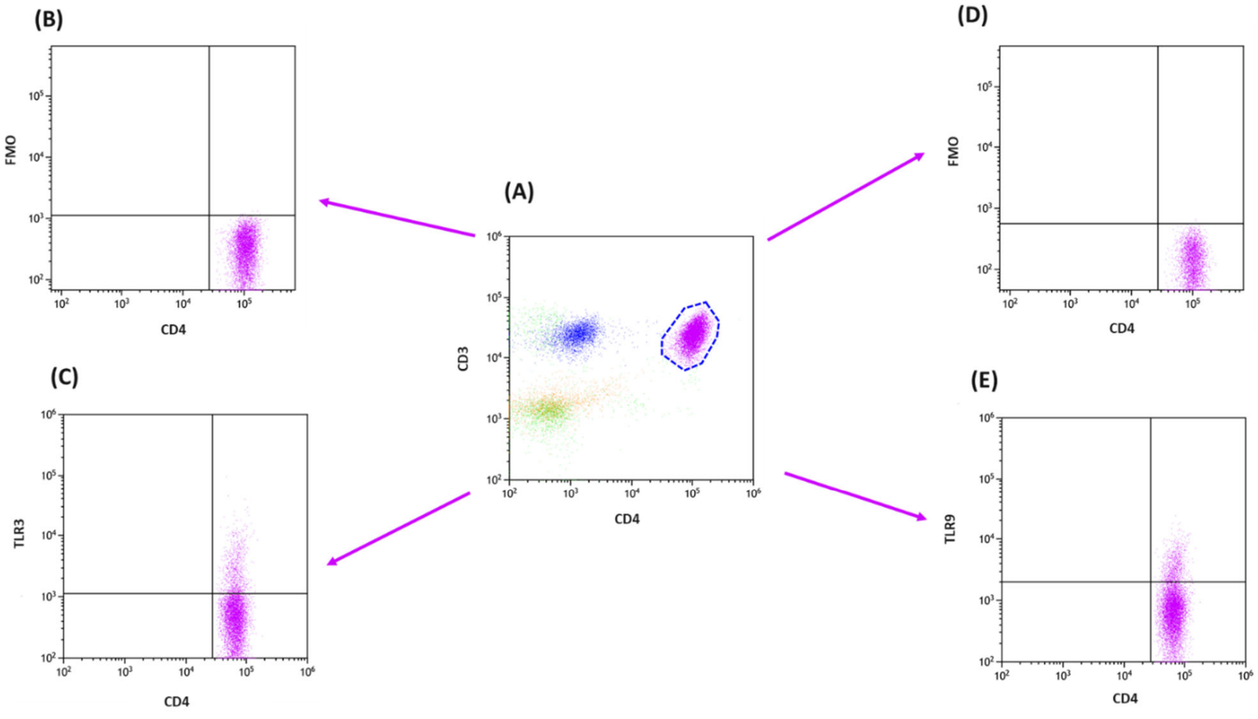

Supplementary Materials Figure S4. The figure shows the analysis of TLR3 and TLR9 expression on CD4$^+$ T cells by flow cytometry using FMO control. Panel (A) shows the gating of the CD3$^+$CD4$^+$ subpopulation, which served as a starting point for further analysis of Toll-like receptor expression. Panels (B) and (D) show FMO (fluorescence minus one) controls for TLR3 (PE) and TLR9 (APC) detection channels, respectively, which allow precise determination of fluorescence signal positivity thresholds by eliminating the influence of autofluorescence and nonspecific background. Panel (C) shows the detected expression of TLR3, while panel (E) shows

the expression of TLR9 on the surface of CD4⁺ T cells. The use of separate FMO controls for each fluorescence channel allowed for reliable and reproducible determination of the percentage of cells positive for a given receptor, increasing the accuracy of the analysis and the correct interpretation of results.

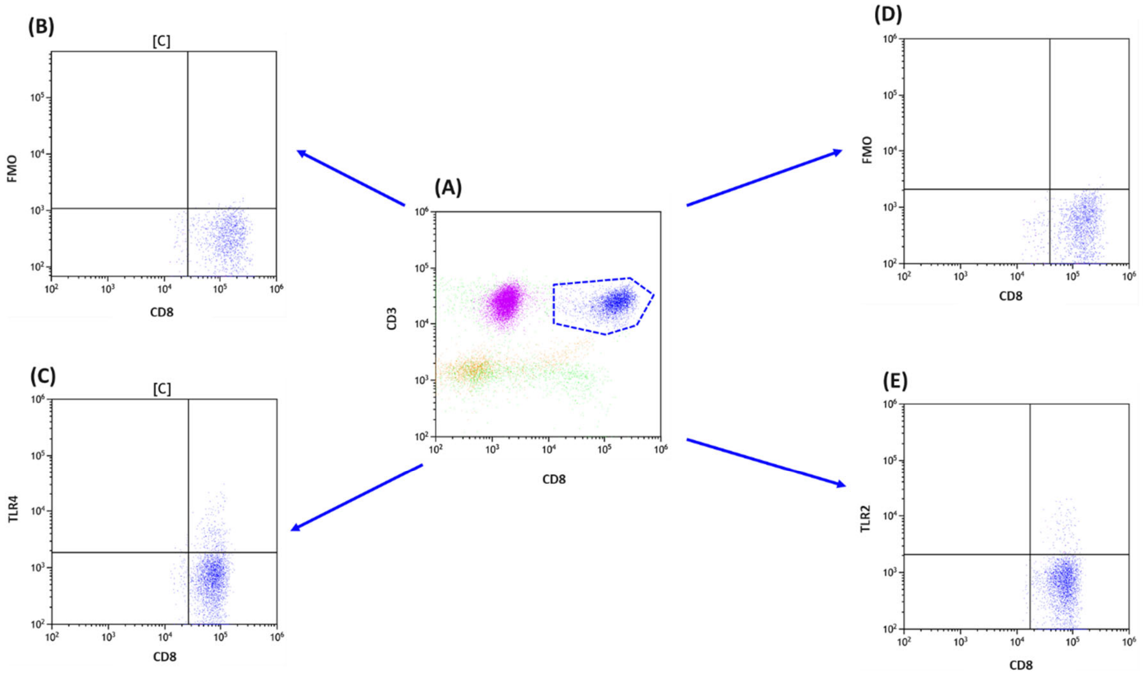

Supplementary Materials Figure S5. The figure shows the analysis of TLR2 and TLR4 receptor expression on CD8⁺ T cells using flow cytometry, including FMO control. Panel (A) shows gating of CD3⁺CD8⁺ cells, which were the starting population for further analysis of Toll-like receptor expression. Panels (B) and (D), respectively, show FMO controls for TLR4 (APC) and TLR2 (PE) fluorescence channels, used to determine signal positivity thresholds by excluding nonspecific background and autofluorescence. Panel (C) shows the detected TLR4 expression, while panel (E) shows TLR2 expression on CD8⁺ cells. The use of FMO control allows for precise and reproducible determination of fluorescence gate boundaries for each receptor, which is crucial for reliable assessment of their presence on the analyzed effector cells of the immune system.

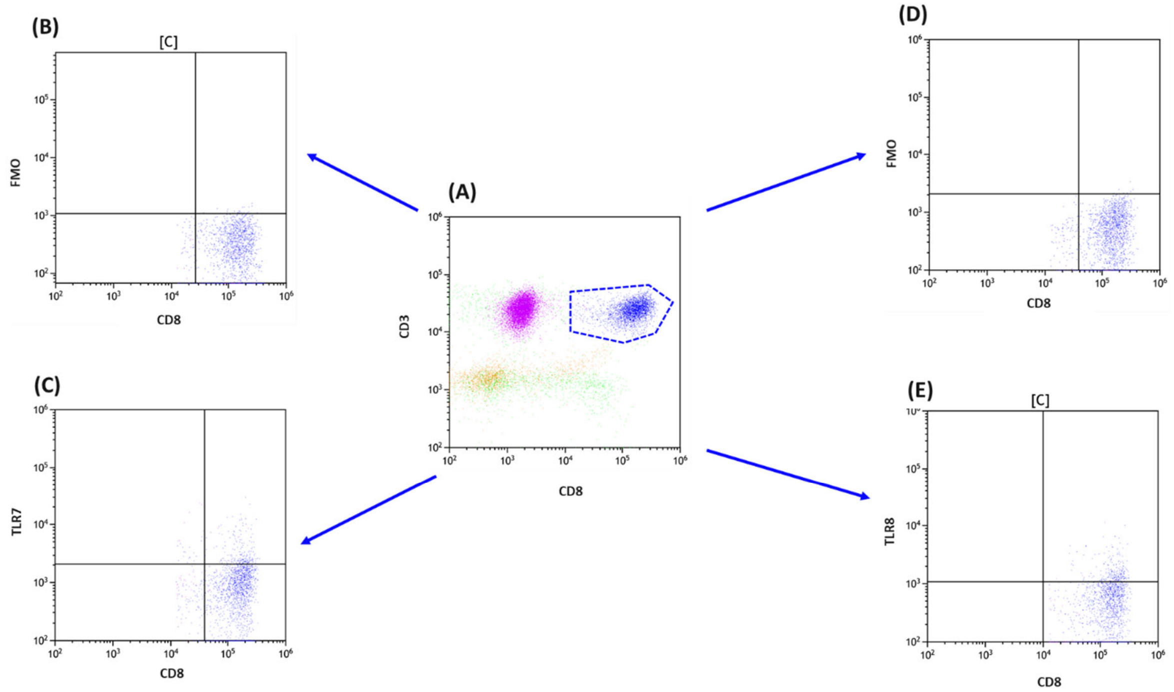

Supplementary Materials Figure S6. The figure shows the analysis of TLR7 and TLR8 expression on CD8⁺ T cells using flow cytometry and the appropriate FMO controls. Panel (A) shows the gating of the CD3⁺CD8⁺ cell population, which was the basis for further analysis. Panels (B) and (D) show FMO controls for the appropriate fluorescence channels, allowing precise determination

of TLR7 and TLR8 positivity thresholds by eliminating background signal and autofluorescence. Panel (C) shows the detected TLR7 expression, while panel (E) shows TLR8 expression on CD8+ T cells. The use of FMO controls allows for a reliable determination of which cells actually express the tested receptors, thus ensuring the accuracy and reproducibility of the flow cytometric analysis.

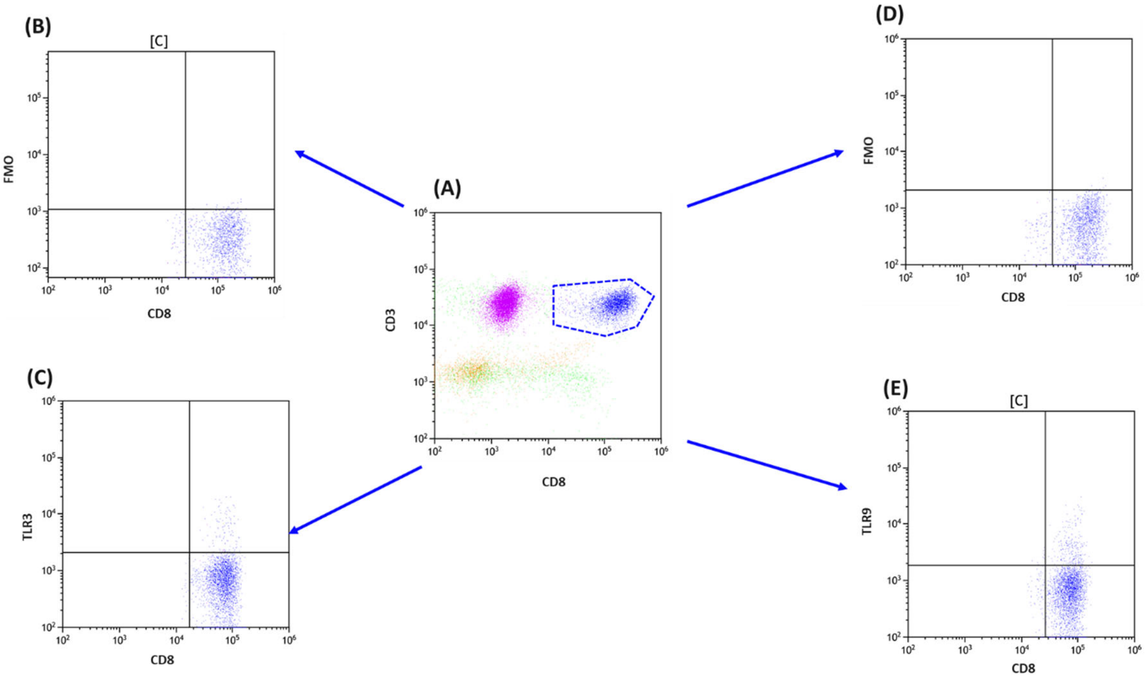

Supplementary Materials Figure S7. The figure shows the analysis of TLR3 and TLR9 expression on CD8+ T cells using flow cytometry. Panel (A) shows the CD3+CD8+ cell population that was isolated for further analysis. Panels (B) and (D) show the FMO (fluorescence minus one) controls for TLR3 and TLR9 channels, respectively, which allow for determining the correct threshold of positivity by eliminating background signal and autofluorescence. Panel (C) shows TLR3 receptor expression, and panel (E) shows TLR9 receptor expression in CD8+ cells. The use of two independent FMO controls for each detection channel allows for precise determination of the range of positivity and ensures the reliability and reproducibility of the analysis of Toll-like receptor expression on cytotoxic lymphocytes.

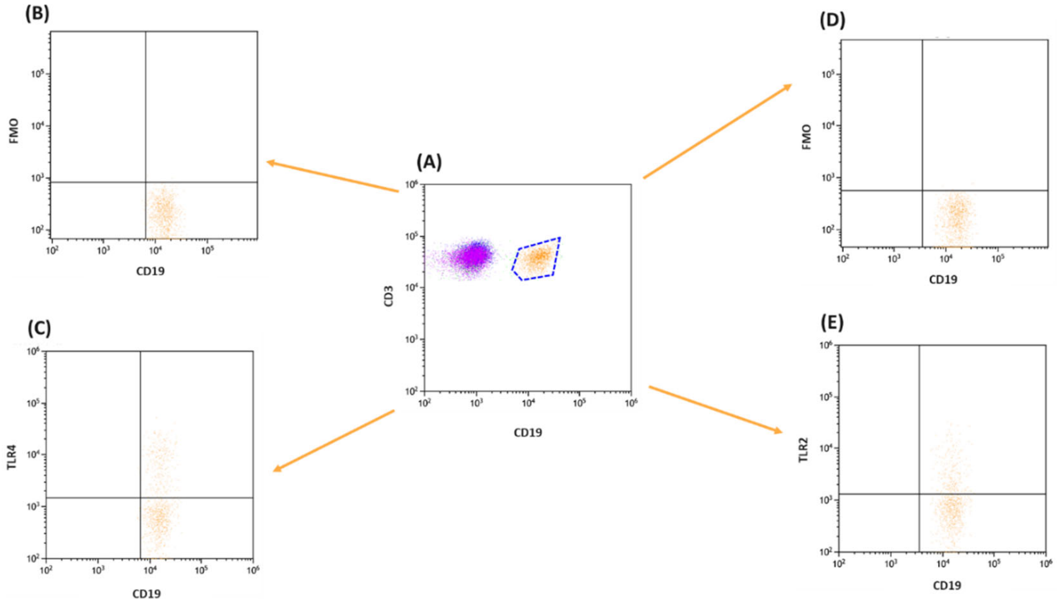

Supplementary Materials Figure S8. The figure shows the analysis of TLR2 and TLR4 expression on B cells (CD19+) using flow cytometry. Panel (A) shows gating of CD3-CD19+ lymphocytes, which were isolated for further analysis. In panels (B) and (D) are included appropriate FMO (fluorescence minus one) controls, allowing for determining the borderline of signal positivity for TLR2

and TLR4, taking into account autofluorescence and background. Panel (C) shows TLR4 expression, and panel (E) shows TLR2 expression within selected B lymphocytes. The use of two independent FMO controls allows for reliable and precise analysis of the expression level of the tested Toll-like receptors, eliminating the risk of false positive results.

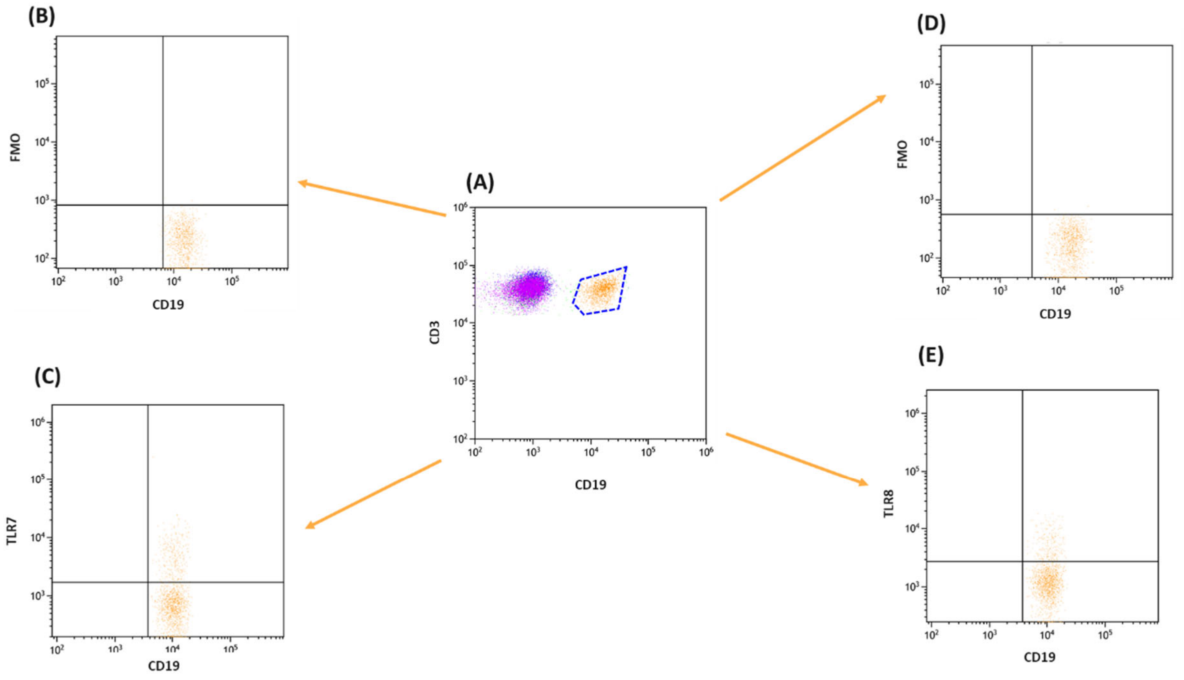

Supplementary Materials Figure S9. Na przedstawionej rycinie zaprezentowano analizę ekspresji receptorów TLR7 i TLR8 na limfocytach B (CD19⁺) metodą cytometrii przepływowej. W panelu (A) pokazano bramkowanie komórek CD3⁻CD19⁺, które zostały wyizolowane do dalszej analizy ekspresji TLR. Panele (B) i (D) zawierają odpowiednie kontrole FMO (fluorescence minus one) umożliwiające precyzyjne ustalenie progów pozytywności fluorescencji dla TLR7 i TLR8, z uwzględnieniem autofluorescencji i sygnału tła. Panel (C) przedstawia poziom ekspresji TLR7, natomiast panel (E) dotyczy ekspresji TLR8 na limfocytach B. Zastosowanie indywidualnych kontroli FMO dla każdego fluorochromu zapewnia wiarygodność uzyskanych wyników, pozwalając na rzetelną ocenę obecności receptorów TLR7 i TLR8 na komórkach CD19⁺.

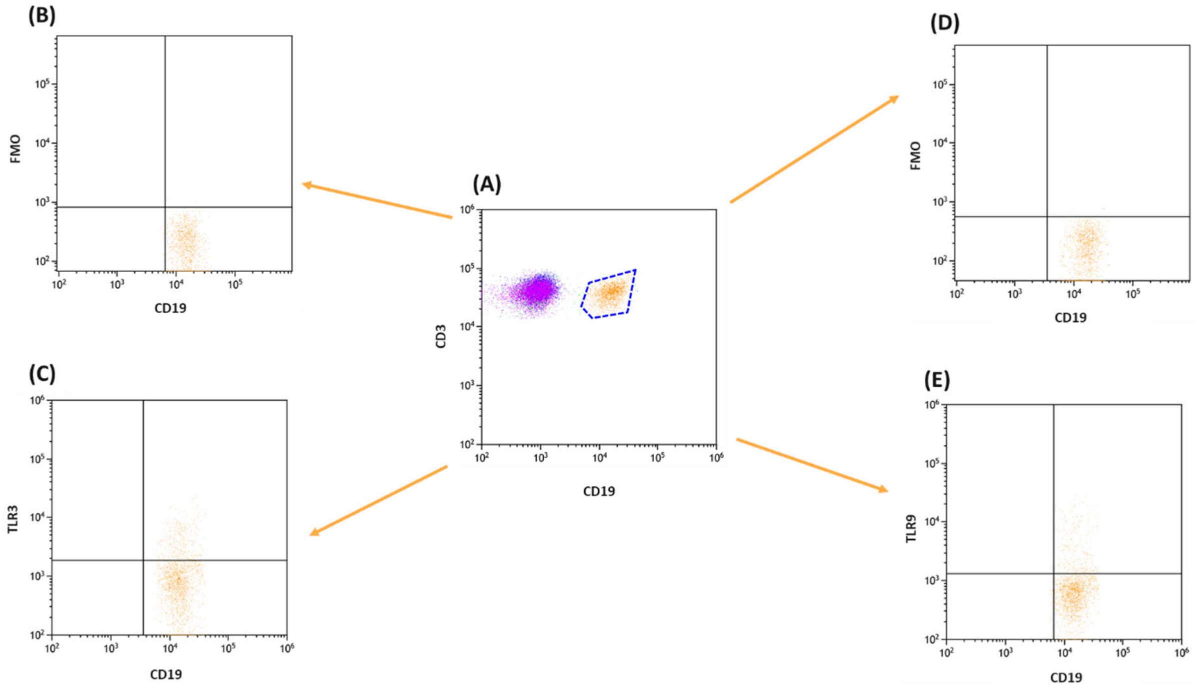

Supplementary Materials Figure S10. The figure shows the analysis of TLR3 and TLR9 expression on the surface of B lymphocytes (CD19⁺) using flow cytometry. The middle panel (A) shows the gating of the CD3⁻CD19⁺ cell population, which was selected for further analysis. Panels (B) and (D) contain FMO (fluorescence minus one) controls, which allow for precise determination of

fluorescence thresholds for the appropriate markers, eliminating the influence of autofluorescence and enabling correct setting of gates for detection of the specific signal. Panel (C) shows the level of TLR3 receptor expression, while panel (E) shows the level of TLR9 receptor expression in the selected CD19$^+$ cell population. The use of appropriate controls and uniform analysis conditions allows for a reliable assessment of the presence of the analyzed receptors in the B lymphocyte subpopulation.

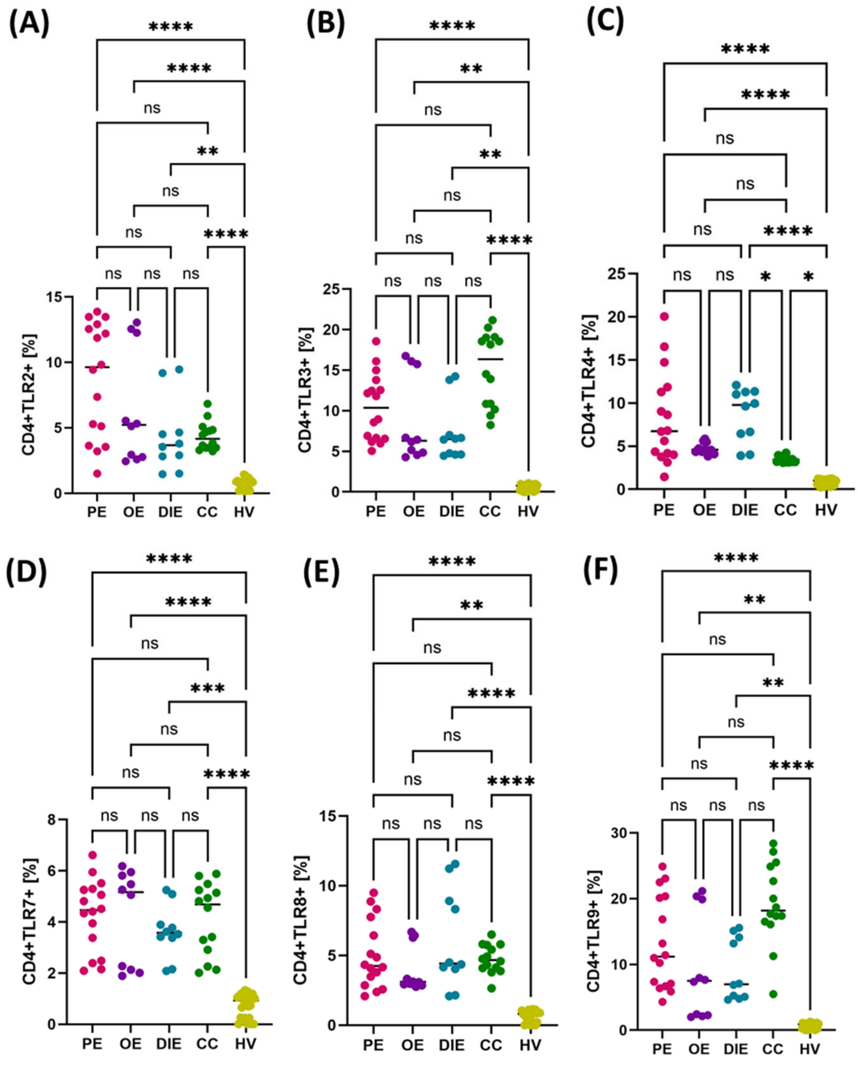

Supplementary Materials Figure S11. Percentage of T helper lymphocytes (CD4$^+$) expressing selected Toll-like receptors: TLR2 (A), TLR3 (B), TLR4 (C), TLR7 (D), TLR8 (E) and TLR9 (F) in individual subtypes of endometriosis — peritoneal (PE), ovarian (OE), deeply infiltrating (DIE), in the cesarean section scar (CC) — and in healthy women (HV). Statistical significance values were marked as follows: $p \leq 0.05$ (*), $p \leq 0.01$ (**), $p \leq 0.001$ (****), $p \leq 0.0001$ (****), ns – no statistical significance.

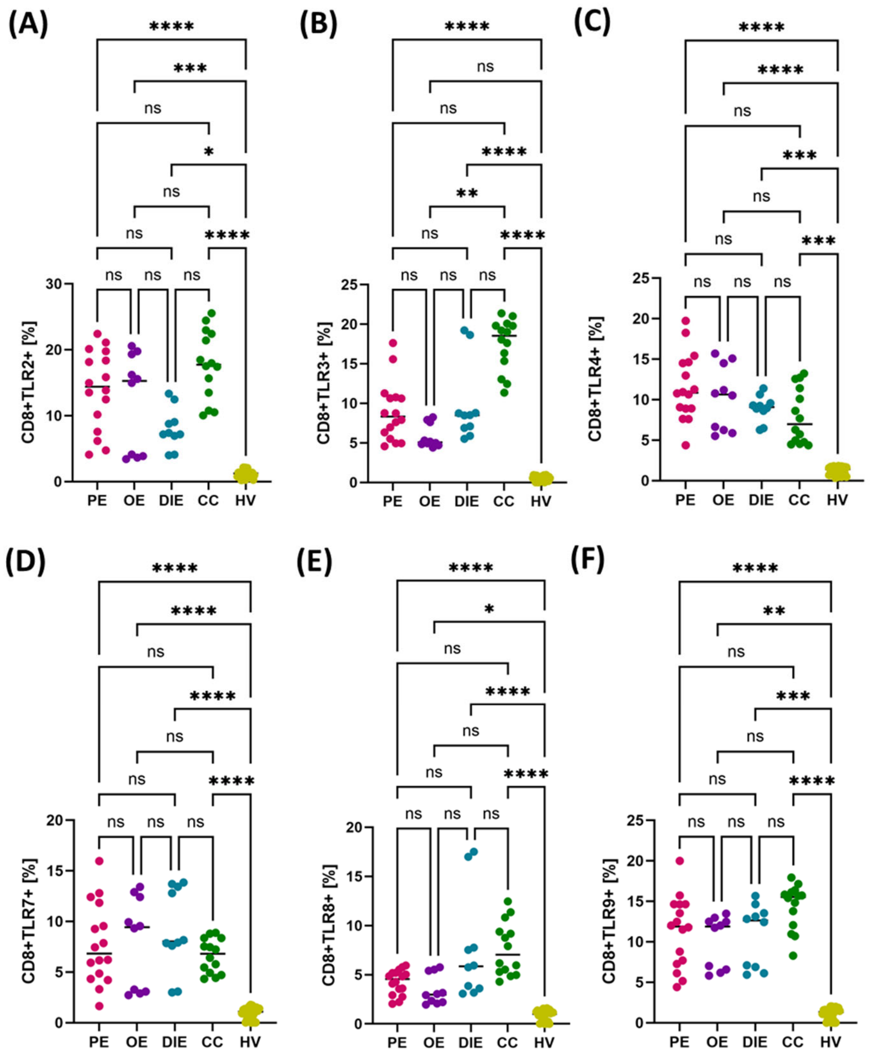

Supplementary Materials Figure S12. Percentage of cytotoxic T lymphocytes ($CD8^+$) expressing selected Toll-like receptors: TLR2 (A), TLR3 (B), TLR4 (C), TLR7 (D), TLR8 (E) and TLR9 (F) in patients with various forms of endometriosis — peritoneal (PE), ovarian (OE), deeply infiltrating (DIE), endometriosis in the cesarean section scar (CC) — compared to the group of healthy women (HV). Statistical significance values were marked as follows: $p \leq 0.05$ (*), $p \leq 0.01$ (**), $p \leq 0.001$ (****), $p \leq 0.0001$ (****), ns – no statistical significance.

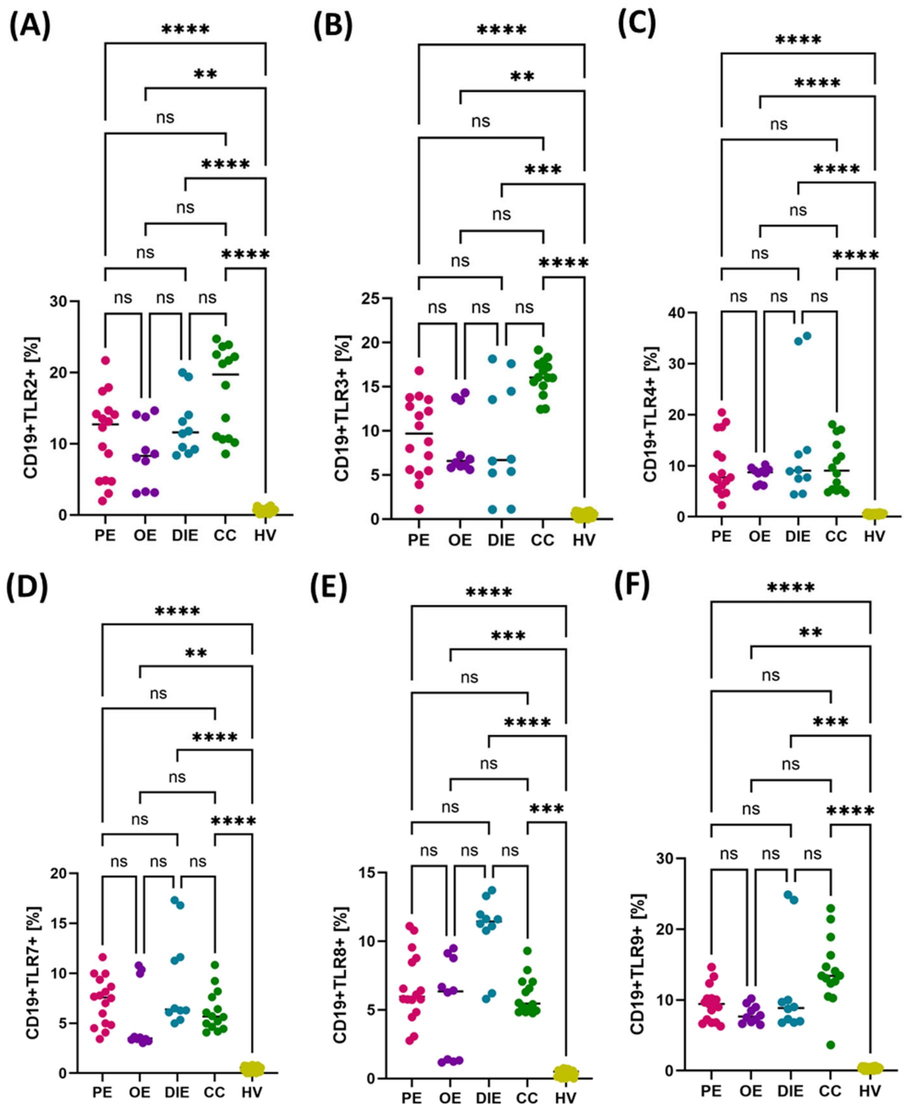

Supplementary Materials Figure S13. Percentage of B lymphocytes (CD19+) expressing selected Toll-like receptors: TLR2 (A), TLR3 (B), TLR4 (C), TLR7 (D), TLR8 (E) and TLR9 (F) in patients with various forms of endometriosis — peritoneal (PE), ovarian (OE), deeply infiltrating (DIE), endometriosis in the cesarean section scar (CC) — compared to the group of healthy women (HV). Statistical significance values were marked as follows: $p \le 0.05$ (*), $p \le 0.01$ (**), $p \le 0.001$ (****), $p \le 0.0001$ (****), ns – no statistical significance.
